# A single-cell survey of cellular hierarchy in acute myeloid leukemia

**DOI:** 10.1186/s13045-020-00941-y

**Published:** 2020-09-25

**Authors:** Junqing Wu, Yanyu Xiao, Jie Sun, Huiyu Sun, Haide Chen, Yuanyuan Zhu, Huarui Fu, Chengxuan Yu, Weigao E., Shujing Lai, Lifeng Ma, Jiaqi Li, Lijiang Fei, Mengmeng Jiang, Jingjing Wang, Fang Ye, Renying Wang, Ziming Zhou, Guodong Zhang, Tingyue Zhang, Qiong Ding, Zou Wang, Sheng Hao, Lizhen Liu, Weiyan Zheng, Jingsong He, Weijia Huang, Yungui Wang, Jin Xie, Tiefeng Li, Tao Cheng, Xiaoping Han, He Huang, Guoji Guo

**Affiliations:** 1 Center for Stem Cell and Regenerative Medicine, The First Affiliated Hospital, Zhejiang University School of Medicine, Hangzhou, 310058 China; 2 Institute of Hematology, The First Affiliated Hospital, Zhejiang University School of Medicine, Hangzhou, 310003 China; 3 Bone Marrow Transplantation Center, The First Affiliated Hospital, Zhejiang University School of Medicine, Hangzhou, 310003 China; 4 Stem Cell Institute, Zhejiang University, Hangzhou, 310058 China; 5 Wuhan Biobank Co., LTD, Wuhan, 430075 China; 6 State Key Laboratory of Fluid Power and Mechatronic Systems, Zhejiang University, Hangzhou, 310058 China; 7 Institute of Applied Mechanics, Zhejiang University, Hangzhou, 310027 China; 8 Institute of Hematology and Blood Disease Hospital, Chinese Academy of Medical Sciences and Peking Union Medical College, Tianjin, 300000 China; 9 Alliance for Atlas of Blood Cells, Tianjin, China; 10 Zhejiang Laboratory for Systems & Precision Medicine, Zhejiang University Medical Center, Hangzhou, 311121 China

**Keywords:** Acute myeloid leukemia, Single-cell mRNA sequencing, Microwell-seq, Ribosomal protein, Single-molecule real-time sequencing, Cancer attractor

## Abstract

**Background:**

Acute myeloid leukemia (AML) is a fatal hematopoietic malignancy and has a prognosis that varies with its genetic complexity. However, there has been no appropriate integrative analysis on the hierarchy of different AML subtypes.

**Methods:**

Using Microwell-seq, a high-throughput single-cell mRNA sequencing platform, we analyzed the cellular hierarchy of bone marrow samples from 40 patients and 3 healthy donors. We also used single-cell single-molecule real-time (SMRT) sequencing to investigate the clonal heterogeneity of AML cells.

**Results:**

From the integrative analysis of 191727 AML cells, we established a single-cell AML landscape and identified an AML progenitor cell cluster with novel AML markers. Patients with ribosomal protein high progenitor cells had a low remission rate. We deduced two types of AML with diverse clinical outcomes. We traced mitochondrial mutations in the AML landscape by combining Microwell-seq with SMRT sequencing. We propose the existence of a phenotypic “cancer attractor” that might help to define a common phenotype for AML progenitor cells. Finally, we explored the potential drug targets by making comparisons between the AML landscape and the Human Cell Landscape.

**Conclusions:**

We identified a key AML progenitor cell cluster. A high ribosomal protein gene level indicates the poor prognosis. We deduced two types of AML and explored the potential drug targets. Our results suggest the existence of a cancer attractor.

## Introduction

Acute myeloid leukemia (AML) is a hematopoietic malignancy with recurrent genetic abnormalities [1, 2]. New therapeutic options such as targeted therapies and monoclonal antibodies may improve the long-term survival in patients with AML [3, 4]. However, the prognosis of AML remains poor in some patients, suggesting its genetic and cellular complexity [5–7]. Therefore, it is of great importance to understand the major hierarchy and cellular compositions in different individuals with AML.

Flow cytometry is widely used for exploring cell heterogeneity in leukemia; however, it is limited to the choice of surface markers [8]. Bulk population sequencing can probe into the cell genome and transcriptome, but misses the information of individual cells. Moreover, integrative analyses of samples from different patients with leukemia prove difficult, due to a lack of assay consistency and precision. The advances in single-cell techniques have made systematic analyses of leukemia cells possible [9, 10]. Several studies have applied single-cell analysis to normal and malignant hematopoietic cells [11–13]. However, because of the limited scales and technical consistency in these studies, an overall picture of AML and the common hierarchy among different patients have not yet been described.

One hallmark of cancer is the reprogramming of energy metabolism to fuel cell growth and division [14]. Ribosome biogenesis is an energy-demanding process, and it has been proposed that ribosomal proteins (RPs) have an effect on tumorigenesis [15]. A previous study reported that RPs exhibited strong dysregulation in particular cancer types, such as breast cancer, melanoma, and thyroid carcinoma [16]. Some RPs are involved in the specification of hematopoietic lineages, and their alterations lead to hematologic disorders, like Diamond-Blackfan anemia, Chromosome 5q deletion syndrome, and Shwachman-Diamond syndrome [17, 18]. However, there is a lack of knowledge on the dysregulation of RPs in AML.

Mitochondrial mutations can suggest clonal relationships [19]. They may preserve information about cell lineage relationships at single-cell resolution [20]. However, no study has examined single-cell mitochondrial mutations in AML to explore the relationship between clonotype and phenotype.

Herein using Microwell-seq, we analyzed 191727 single cells of bone marrow mononuclear cells (BMMCs) from 40 de novo AMLs and 8561 single cells of BMMCs from three normal donors [21]. To investigate the cellular and molecular changes after AML treatment, we followed-up four patients after they received chemotherapy. We demonstrated a global transcriptional heterogeneity and a lack of clear cell fate boundaries in AML samples. We showed that an AML progenitor cell cluster was associated with a dysregulation of RPs and revealed that patients with RP high progenitor cells had a low remission rate. We deduced two types of AML with diverse clinical outcomes. We suggested the existence of a phenotypic “cancer attractor” that might help to define a common phenotype for AML progenitor cells by combining Microwell-seq with SMRT sequencing. Finally, we investigated the potential targets by making comparisons with the Human Cell Landscape. These datasets have deepened our understanding and might open a way for novel diagnostic and therapeutic strategies in AML.

## Results

### Analysis of normal BMMC hierarchy

**Fig. 1:**
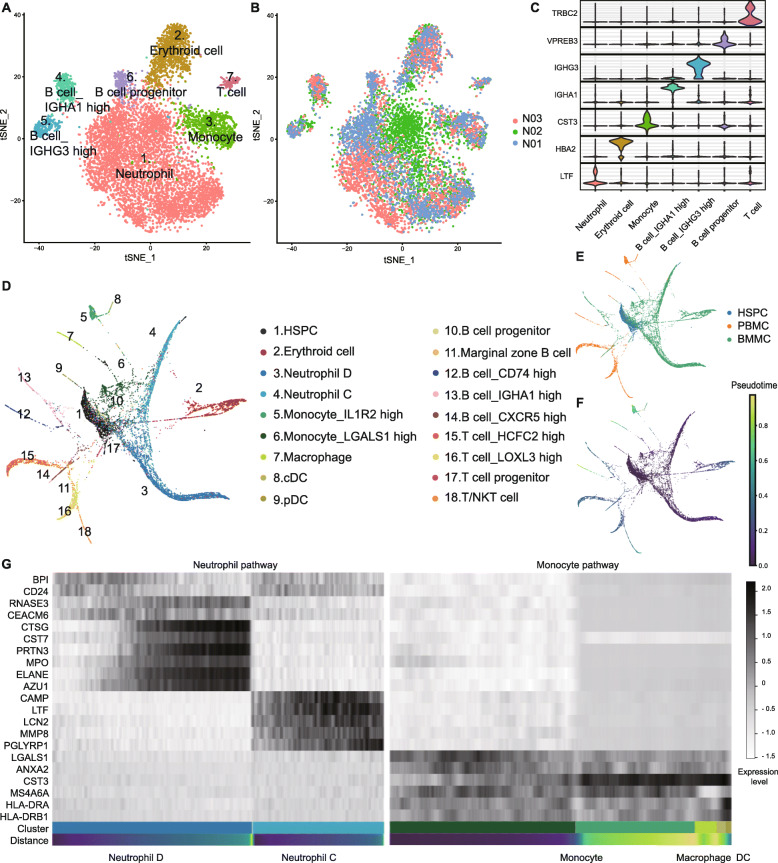
Analysis of normal BMMC hierarchy. **a**, **b** t-SNE analysis of normal BMMCs. Clusters and individuals are labeled in different colors and numbers. **c** Violin plots of differentially expressed genes. The horizontal axis shows the clusters. **d**, **e** PAGA analysis of normal BMMCs, PBMCs, and HSPCs. Clusters and samples are labeled in different colors and numbers. **f** Trajectory analysis of BMMCs, PBMCs, and HSPCs. **g** Heatmap of marker genes in neutrophil and monocyte pathways. Black and white represent high and low expression levels, respectively

To gain insights into the heterogeneity of normal and malignant hematopoiesis, we first profiled the heterogeneity in normal BMMCs. We used Microwell-seq on three healthy donors and established the analysis pipeline (Fig. S1A) [21]. We performed t-Distributed stochastic neighbor embedding (t-SNE) analysis of individuals (Fig. S1B and Supplementary Table 1). The t-SNE map of 8561 normal BMMCs of three healthy donors is shown in Fig. 1a, b. According to the gene expression patterns, we identified lymphoid, erythroid, and myeloid lineages (Fig. 1a, c and Supplementary Table 2) [22, 23]. Neutrophils are divided into three main types, neutrophil A, B, and C, along with three extended types, neutrophil D, E, and F (Fig. S2A and Supplementary Table 2). The related marker genes are shown in Fig. S2B, C.

To perform lineage trajectory analyses, we integrated another 2000 hematopoietic stem/progenitor cells (HSPCs) and 2719 peripheral blood mononuclear cells (PBMCs) from our previous study to get a total of 13280 healthy cells [24]. Using partition-based graph abstraction (PAGA), we revealed distinct developmental branches and built a transcriptional landscape for normal human hematopoiesis (Fig. 1d-f and Supplementary Table 3). The expression levels of marker genes change in the myeloid path, in conformity to the t-SNE analyses above (Fig. 1g).

### Identifying the progenitor cell cluster of de novo AMLs

**Fig. 2:**
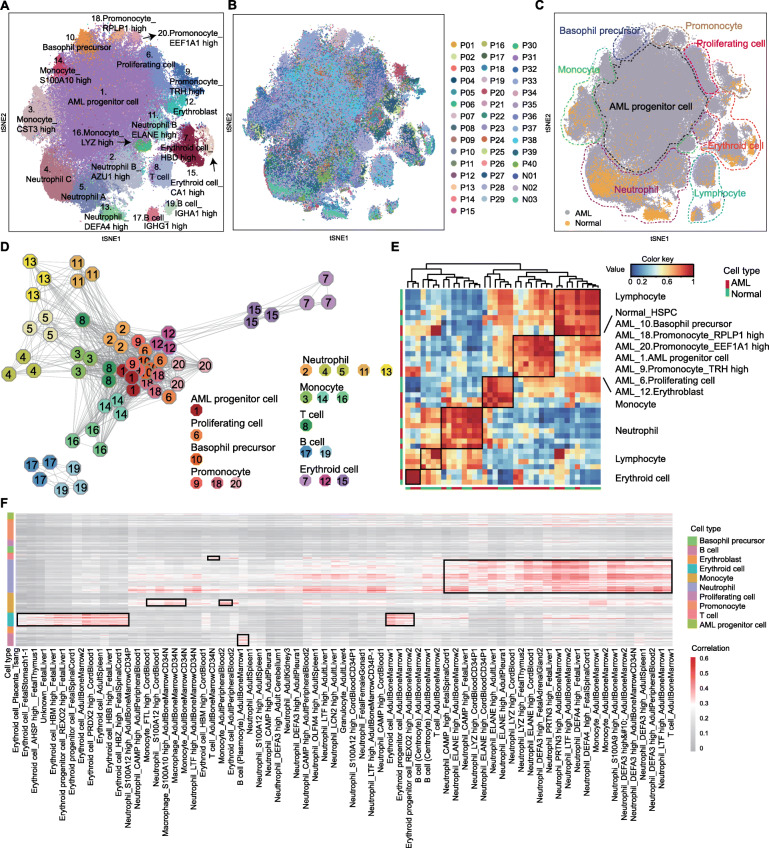
Identifying the progenitor cell cluster of de novo AMLs. **a**, **b** t-SNE analysis of AML and normal BMMCs. Twenty clusters, 40 patients, and 3 normal donors are labeled in different colors and numbers. **c** t-SNE analysis of BMMCs. AML and normal cells are labeled in different colors. Different cell types are surrounded by dotted lines. **d** Connection network map of the clusters. Each cluster is represented by three octagons. Clusters correspond to those in **a**. **e** Correlation matrix of clusters in normal and AML cells. Red and blue represent high and low correlations, respectively. The *X-* and *Y*-axis represent the AML and normal cell. **f** Single-cell blast results of AML cells. Each row represents cells in AML. Each column represents one cell type in HCL reference. The length of cell type bar represents the cluster number. Red and gray represent high and low correlations, respectively

We then moved on to understand the cellular hierarchy in AMLs. Forty newly diagnosed patients were recruited for Microwell-seq analysis (Supplementary Table 4). AML cell groups were less distinct when compared with those of healthy bone marrow. The presence of significant transcriptome variation and lack of cell cluster boundaries were the most commonly shared phenotypes among single-cell data from different patients (Fig. S3, S4). To gain an integrative view of the different AML samples, we removed the batch effect and generated an overall t-SNE map with 40 patients and 3 healthy donors (Fig. 2a, b, S5A, and Supplementary Table 5). The AML cell landscape contains 20 clusters. The clusters of neutrophil, monocyte, erythroid cells, and lymphoid cells were shared by both normal and AML BMMCs. However, there was a cloudy cluster at the center with no functional maker genes (Fig. 2c).

The genetic network demonstrates that this cluster has a close relationship with myeloid cells (Fig. 2d), and the correlation analysis suggests a resemblance to HSPC (Fig. 2e). Therefore, we named it AML progenitor cell cluster. Using a single-cell mapping pipeline, we estimated its similarity to immune cells in the Human Cell Landscape (HCL) (http://bis.zju.edu.cn/HCL/index.html) [25]. Unlike the normal cells in Fig. S5B, the AML progenitor cell cluster was not able to match any normal human hematopoietic or non-hematopoietic cells (Fig. 2 f). This phenomena was also obvious when taking the normal BMMCs as individual references (Fig. S5C, D).

### Characterizing the single-cell gene expression patterns of AML progenitor cell cluster

**Fig. 3:**
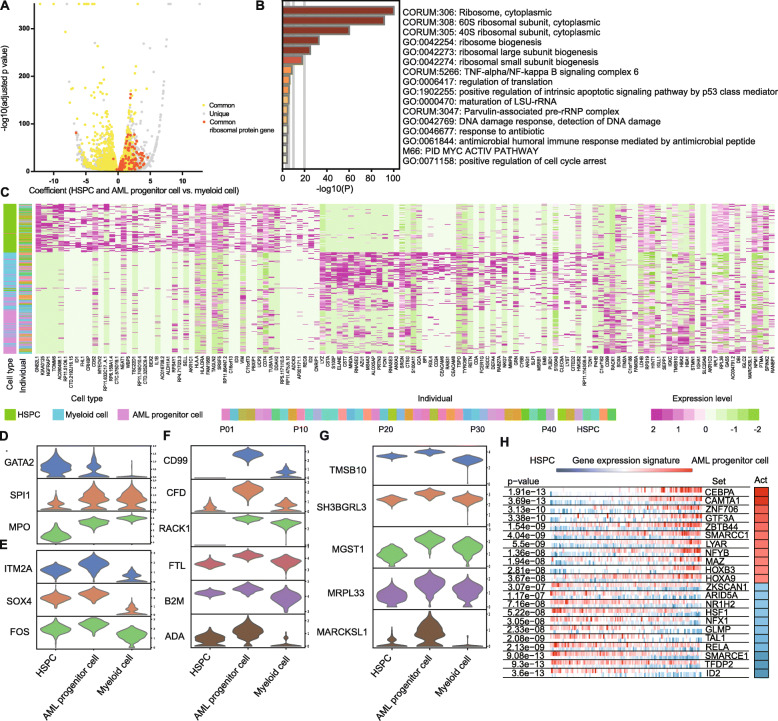
Characterizing the single-cell gene expression patterns of AML progenitor cell cluster. **a** Volcano plot of DEGs between progenitor cells (HSPCs and AML progenitor cells) and myeloid cells. Yellow represents the common genes shared by both HSPCs and AML progenitor cells. Gray represents the unique genes in HSPCs or AML progenitor cells. Orange represents the common ribosomal protein (RP) genes shared by both HSPCs and AML progenitor cells. The dots on the right represent the higher expressed genes in progenitor cells, and those on the left represent lower. **b** Metascape GO analysis for viewing top enrichment terms in AML progenitor cells. Color shows the *p* value. **c** Heat map of top DEGs among HSPCs, AML progenitor cells, and myeloid cells. Cell type and individual are indicated by the colored bars. Individual includes the AML patients and HSPC donors. **d**–**g** Violin plots of DEGs among HSPCs, AML progenitor cells, and myeloid cells. The genes are related to hematopoietic development (**d**), primitive state (**e**), AML (**f**), and other solid tumors (**g**) in previous studies. **h** VIPER plot of activated (red) and repressed (blue) TFs in AML progenitor cells. The gene expression signature is rank-sorted from the one most downregulated to the one most upregulated in the AML progenitor cells vs. HSPCs. The column on the right shows the activity level

Since the AML progenitor cells were similar to the HSPCs, we further used the volcano plot to explore the genes with similar expression levels. When compared to the myeloid cells, AML progenitor cells and HSPCs had many upregulated genes in common, especially the ribosomal protein (RP) genes (Fig. 3a and Supplementary Table 6). To confirm our results, we compared three projects of the Cancer Genome Atlas (TCGA) containing AMLs and one Gene Expression Omnibus (GEO) series containing normal samples. Among the RP genes detected in our study and public databases, the expression levels of 71 RP genes were higher in at least 1 TCGA project, and 53 RP genes were higher in all the 3 TCGA projects (Fig. S6a). Using Metascape analysis, we illustrated that AML progenitor cells had a high transcription activity (Fig. 3b, Fig. S7A).

Despite this similarity, there are also differentially expressed genes (DEGs) among HSPCs, AML progenitor cells and myeloid cells (Fig. 3c). The gene expression patterns revealed that the AML progenitor cells were in the intermediate state from HSPCs to differentiated myeloid cells. Specifically, the expression levels of GATA2, SPI1 (PU.1), and MPO in AML progenitor cells were in the middle (Fig. 3d). GATA2 and SPI1 (PU.1) are key genes in hematopoietic development, and MPO is the myeloid marker gene [26]. However, some genes involved in the primitive state are expressed highest in the AML progenitor cells, such as SOX4, FOS, and ITM2A (Fig. 3e) [27–29]. Further, CD99, CFD, RACK1, FTL, B2M, and ADA are overexpressed in AML progenitor cell cluster, and previous studies found a relationship between these genes and AML (Fig. 3f) [30–35]. The AML progenitor cells also highly expressed genes such as TMSB10, SH3BGRL3, MGST1, MRPL33, and MARCKSL1, which were associated with solid tumors but not previously reported in AML (Fig. 3g) [36–40]. All these highly expressed genes were confirmed by TCGA (Fig. S6B, C).

We performed VIPER (Virtual Inference of Protein-activity by Enriched Regulon) analysis in order to suggest transcription factor (TF) activity in AML progenitor cells [41]. It uses the expression of genes that are regulated by a given TF as an accurate reporter of its activity. Among the top differentially expressed TFs in myeloid cells in comparison to HSPCs, TFs involved in hematopoietic development, such as SPI1, KLF5 and JDP2, were activated (Fig. S7B) [42, 43]. However, TFs involved in malignancy, such as SMARCC1, HOXA9, and HOXB3, were active in AML progenitor cells (Fig. 3h) [44–46].

FLT3 is overexpressed in both mutated and non-mutated malignant cells with FLT3-ITD being one of the most common mutations in AML [26, 47]. We selected the mutated and non-mutated cases of various expression levels and found that FLT3 was mainly accumulated in AML progenitor and proliferating cell clusters (Fig. S7C).

Altogether, we identified a high transcriptional activity progenitor cell group in AMLs and presented cellular hierarchies of BMMCs in healthy and AML state at the single-cell level.

### Intratumoral heterogeneity in AML progenitor cells predicts prognosis

**Fig. 4:**
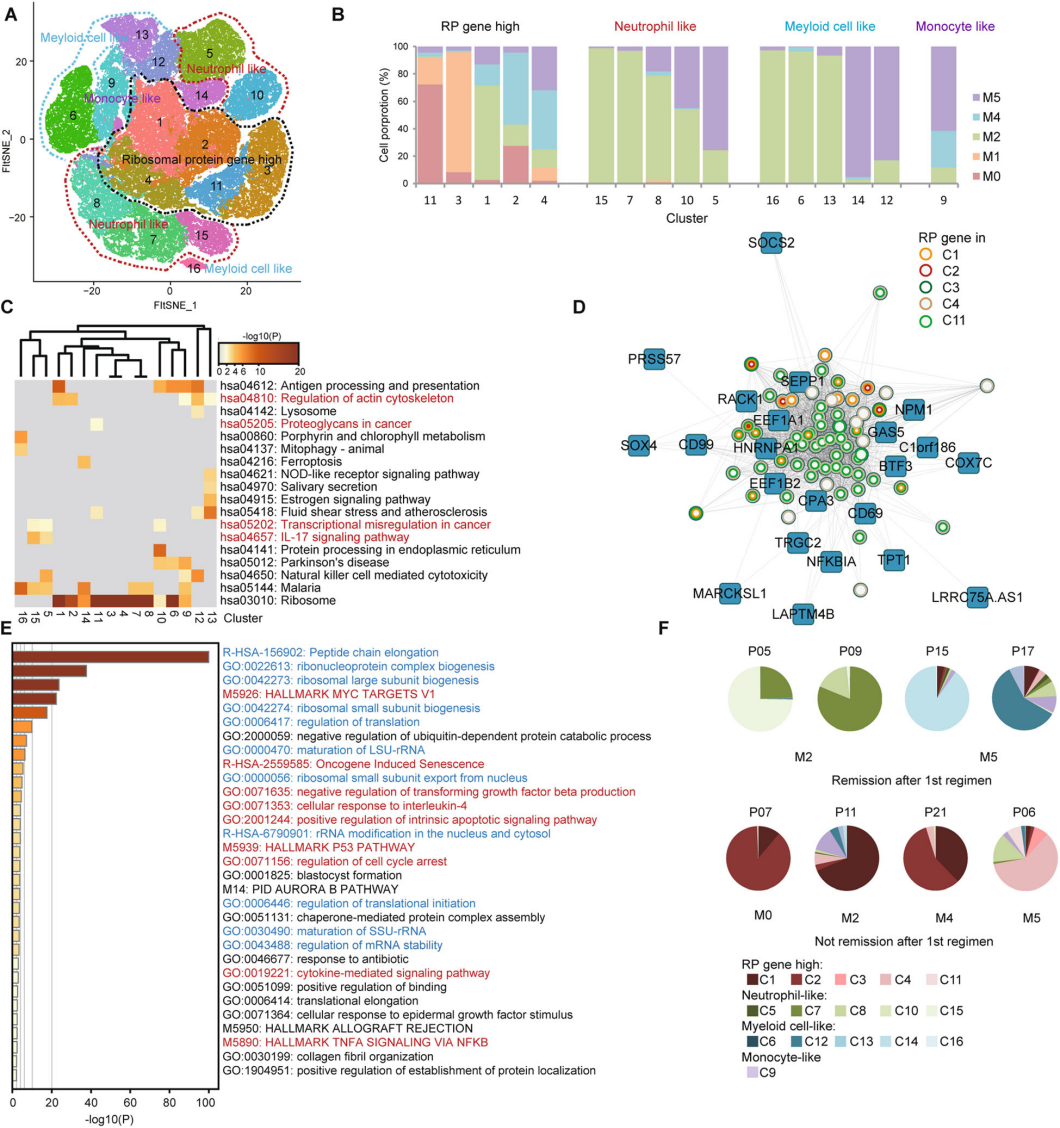
Intratumoral heterogeneity in AML progenitor cells predicts prognosis. **a** Subdivision t-SNE analysis of AML progenitor cells. Sixteen clusters are classified into four groups and are labeled in different colors and numbers. **b** Relative proportion analysis of four cell groups in **a**. Cells of different FAB subtypes are labeled in different colors. **c** Metascape KEGG pathway analysis for viewing enrichment terms in 16 clusters. Color shows the *p* value. **d** Visualization of the network with the genes of top weighted connectivity in the RP gene module. The circles represent the RP genes, while the colors show the genes in different clusters. **e** Metascape GO analysis for viewing top enrichment terms in the RP gene module. Color shows the *p* value. The terms in blue are associated with ribosome biogenesis, and in red are tumor-related. **f** Pie charts displaying cell distribution of AML progenitor cells of 16 clusters belonging to four cell groups. The patient ID is on the top of each chart. The subtype is on the bottom

Despite common characteristics, AML progenitor cells vary among patients (Fig. S5A). We subdivided the common AML progenitor cell cluster to characterize the potential heterogeneity. With respect to the top marker genes, we clustered AML progenitor cell cluster into 16 clusters and summarized them into four groups (Fig. 4a, Supplementary Table 7). C1, 2, 3, 4, and 11 were RP gene high clusters. The rest included neutrophil-like, monocyte-like, and myeloid cell-like clusters (Fig. 4a, b). Generally speaking, our clustering is consistent with the French–American–British (FAB) classification; however, it is more detailed and is better at revealing the functional states of AML progenitor cells based on single-cell transcriptomic data. For example, the cells belonging to M5 patients can be divided into four clusters, C5, C9, C12, and C14 (Fig. S8A).

Enrichment analysis using Metascape analysis revealed that different clusters had different functional preferences. In RP gene high clusters, C1 and C2 were active in regulating the actin cytoskeleton. C4 was related to the P53 pathway. C11 was rich in cancer proteoglycans. In neutrophil-like clusters, C5 and C15 were related to the IL-17 signaling pathway and translational misregulation in cancer. C7 and C8 were rich in MYC targets (Fig. 4c, Fig. S8B, C). Moreover, the cycling cells also varied among the clusters, indicating the different proliferating states (Fig. S8D).

We used the weighted correlation network analysis (WGCNA) to explore the gene regulatory network in AML progenitor cells and identified 18 modules according to the co-expression patterns (Fig. S8E). Among these, one module was dominated by RP genes (Fig. S8F). We presented the network of this module using genes with top weighted connectivity. All the RP genes in this module could be found in RP gene high clusters (Fig. 4d). Besides their role in transcription, the genes in this module were equally strongly related to tumor-associated terms, suggesting their roles in AML progression (Fig. 4e).

We subsequently investigated whether the cell proportion of specific clusters predict prognosis. Twenty-five patients with IA as the first regimen were tracked. Among these, 18 patients were in remission after the first regimen. The AML progenitor cells of 16 out of 18 patients were mostly distributed in neutrophil-like, monocyte-like, and myeloid cell-like clusters. Seven patients of the 25 were not in remission after the first regimen. The AML progenitor cells of four of these seven patients were mostly in the RP gene high clusters (Fig. 4f, Fig. S8G). We supposed that the progenitor cells in RP gene high clusters may possess limited differentiation ability leading to the lower rate of remission. In addition, we summarized a list of DEGs in these clusters (Supplementary Table 8).

We attempted to classify all AML samples from a single-cell perspective. We found that individual t-SNE maps could be grouped into two types based on the proportion of cells in the AML progenitor cluster (C1), with a 50% boundary. In type I, cluster 1 was the largest cluster containing more than 50% of total cells (median proportion, 80.77%, average proportion, 77.81%). Type II patients had smaller C1 (median proportion, 30.50%, average proportion, 31.66%) (Fig. S3, S4 and Supplementary Table 1). Patient 2, 3, 4, 5, 6, 8, 9, 12, 14, 15, 21, 22, 24, 29, 30, and 34 were classified into type I patients, and the rest were type II patients. This grouping appeared to be independent of known AML subtypes (FAB or World Health Organization classification) given that we found both type I and II patients in every AML subtype (Supplementary Table 4) [48, 49].

During prognosis evaluation, we found that the elderly tended to be type I patients, and it seemed more difficult for the type I patients to reach complete remission (CR) (Fig. S8H). Excluding the patients who were lost to follow-up, the CR rate was 55.56% (5/9) for type I, and 84.21% (16/19) for type II after the second regimen (p < 0.05, Hypergeometric test). The 1-year survival rate was 53.3% for type I and 70.0% for type II (Fig. S8I). Although there was no statistical significance (*p* = 0.198, log-rank test), we found that more type I patients died shortly after diagnosis, even before the initiation of treatment.

Altogether, we found that a high expression levels of RPs in AML progenitor cells was a predictor of poor prognosis providing a new perspective for the classification of AML.

### Clinical implication of AML progenitor cells from diagnosis to relapse

The cellular hierarchy of leukemia cells changes from diagnosis to relapse. We applied the Microwell-seq to four individuals both before and after treatment regimens (Fig. S3, S9 and Supplementary Table 1). P-extra 1 and 2 were in CR. The BMMCs of P-extra 1 were collected before the second regimen, while those of P-extra 2 were collected during myelosuppression after the first regimen. P20 was in relapse while P04, with myelomonocytic leukemia, was in partial remission with normal neutrophil and abnormal monocyte levels after the regimen.

Using uniform approximation and projection (UMAP), we visualized cells before and after treatment, and summarized the findings in a bar plot. We took N02 as normal control. Circos plot showed the relationship among different states. Heatmap revealed the highly variable genes.

**Fig. 5:**
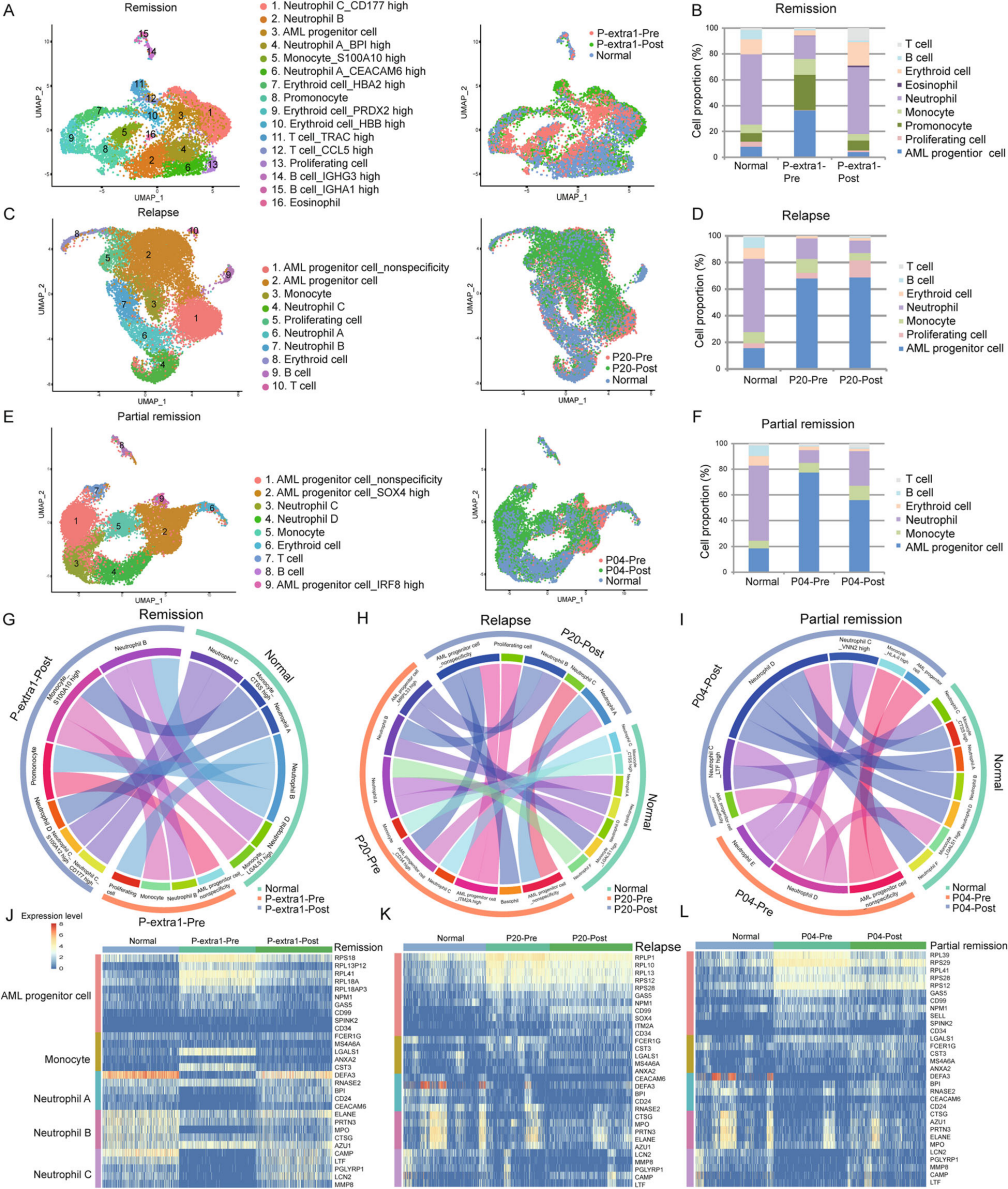
Clinical implication of AML progenitor cells from diagnosis to relapse. **a**, **c**, **e** UMAP analysis of normal and AML individuals before and after treatment regimen. The AML individuals are in remission (P-extra 1, **a**), relapse (P20, **c**), and partial remission (P04, **e**). The normal sample is N02. Colors represent clusters in left panels, and individuals in the right panels. **b**, **d**, **f** Relative proportion analysis of clusters in normal and AML individuals. Cell types are labeled in different colors. **g**–**i** Circos plots showing the correlation of clusters. The similar clusters are connected by lines. **j**–**l** Heatmap of differentially expressed marker genes. Each row represents marker genes of each cell type. Each column represents cells in different status. Red and yellow represents high expression level while blue represents low levels

As in remission, most cells in P-extra 1-post overlap with normal cells (Fig. 5a and Supplementary Table 9). This was equally confirmed by cell number count, as the amount of AML progenitor and proliferating cells reduced, and neutrophils increased (Fig. 5b). The circos plot revealed a correlation among the three states (Fig. 5g). The clusters of myeloid cells at remission were linked to those in normal, while the AML progenitor cells deviated from the normal. Log-normalized data of highly variable genes were plotted on the heatmap. Interestingly, the RP and primitive genes were high at diagnosis, but low after remission. The neutrophil genes showed an opposite trend (Fig. 5j). For P-extra 2, although the myelosuppression led to a high proportion of lymphocytes, the results were similar (Fig. S10).

As in relapse, the progenitor cells of P20-post still dominate the bone marrow (Fig. 5c, d and Supplementary Table 9). In the circos plot, the progenitor cells at relapse were still connected with the same cluster at diagnosis, indicating an abnormality (Fig. 5h). The expression levels of RP and primitive genes in P20-post remained high (Fig. 5k). In addition, we summarized the up- and downregulated genes in AML progenitor cells at relapse (Supplementary Table 10).

As in partial remission, the cells of P04-post were partly mixed with those in normal, and the number of progenitor cells declined slightly (Fig. 5e, f and Supplementary Table 9). In the circos plot, there was a connection between normal and some P04-post clusters (Fig. 5i). The expression levels of RP and primitive genes in P04-post were lower than those in P04-pre (Fig. 5l).

These results indicate that the expression levels of specific genes, especially the RP genes, can be used to evaluate tumor progression. It also proves that Microwell-seq is a useful tool for evaluating curative effects.

### Resolving intratumoral heterogeneity in monocytic leukemia AMLs

We then focused our analysis to one AML subtype, M5, and integrated all the 15 monocytic leukemia data in our study. Although standard induction chemotherapy induces remission in most patients with AML, patients with refractory disease show poor sensitivity to treatment [50]. In our study, P06, P14, and P16 had refractory disease; the rest 12 were non-refractory patients.

The AML progenitor cells varied greatly across patients (Fig. S11A). We randomly selected 12000 cells in refractory and non-refractory patients, and distinguished unique clusters of refractory (C5) and non-refractory (C13) patients (Fig. S11B-E, Supplementary Table 11). Gene set enrichment analysis (GSEA) revealed that MYC, SRC, RELA, the proto-oncogenes, and MTOR were overexpressed in C5 in comparison to C13, suggesting its malignant properties (Fig. S11F). According to the TCGA, high expression levels of SRC and MTOR are predictors of poor prognosis (Fig. S11G). Gene Ontology (GO) analysis using EnrichR revealed other enriched terms in C5 (Fig. S11H and Supplementary Table 12). The TFs, such as CEBPG, GATA2, MAX, and JUN, were active in C5, while CEBPE, GATA1, MAZ, and JUND were active in C13 (Fig. S11I). All these differences could lead to differences in responses to treatment.

### The association of genetic mutation with cellular hierarchy by SMRT sequencing at single-cell level

**Fig. 6:**
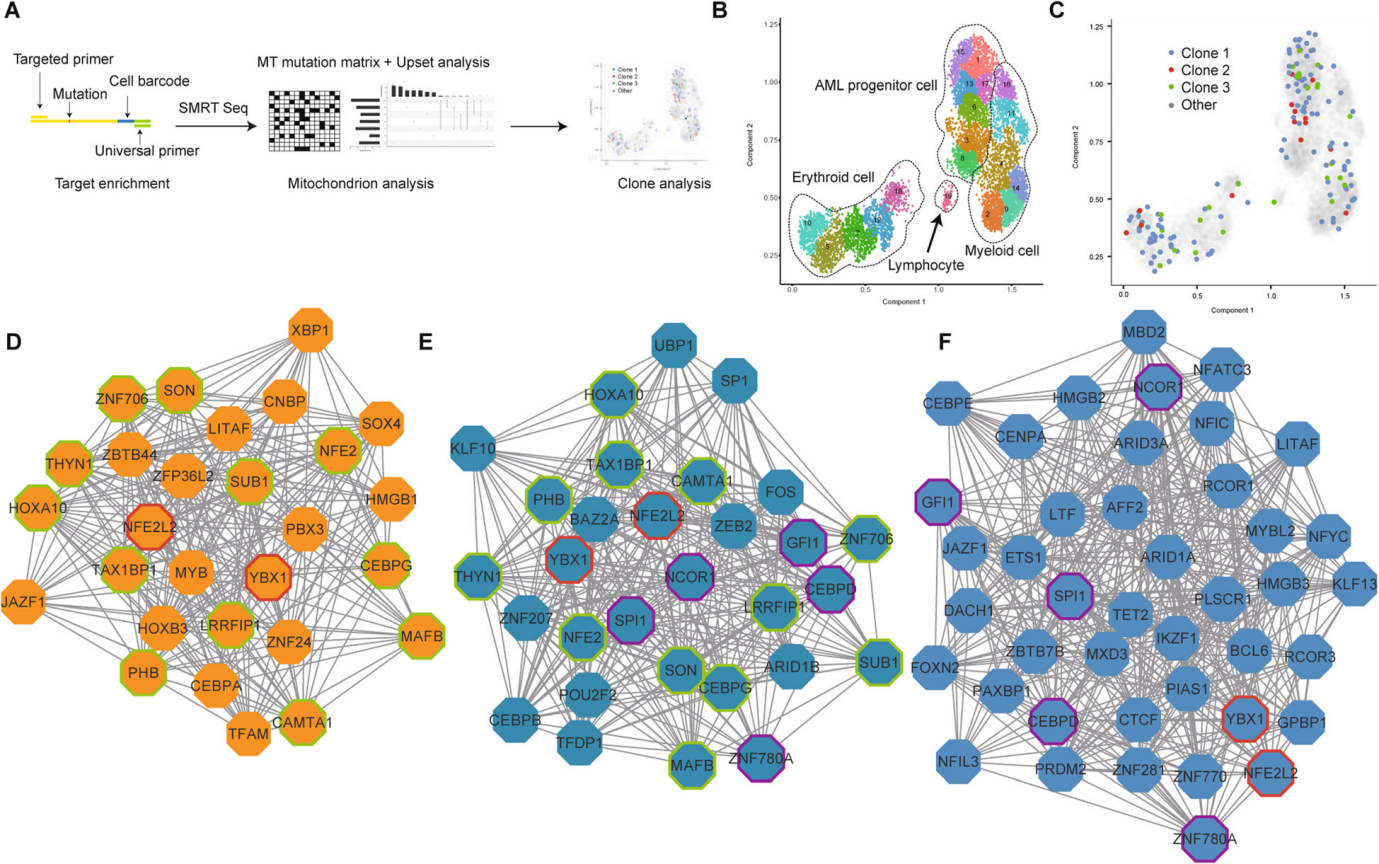
The association of genetic mutation with cellular hierarchy by SMRT sequencing at the single-cell level. **a** Procedures for acquiring mutation information from single cell, and the following analyses. **b** Trajectory analysis of P25 using UMAP/Monocle 3. Clusters are labeled in different colors and numbers. **c** Clone projection of P25 using UMAP/Monocle 3. Clones are labeled in different colors. **d**-**f** Visualization of attractor networks with the core genes of the AML progenitor cells (**d**), AML myeloid cells (**e**), and normal myeloid cells (**f**) in comparison to HSPCs

For the limitation of coverage, the 3′ single-cell transcriptome analysis failed to identify key mutations in leukemia. We, therefore, integrated long-read sequencing with the Microwell-seq experiment. We used the targeted upstream primer and universal downstream primer to amplify specific genes from single-cell cDNA for SMRT sequencing, so that we could get both the mutation sites and cell barcodes (Fig. 6a).

Previous studies reported that mtDNA mutations provide innate and natural barcodes to infer clonal associations [19, 51]. We chose MT-ND4, a mitochondrial gene, for lineage tracing using SMRT sequencing. We identified the heteroplasmic variants of MT-ND4 from single cells of patient P25, P10, and P17. After filtration, we got the average of 3406 reads aligned to the MT-ND4 transcript reference. The average sequencing depth for mutation sites was 3041. Among these, about 711 reads could be projected to the UMAP per sample. We separated these cells into several subclones with respect to their unique mutations (Fig. S12A-C). Monocle3/UMAP was adopted to display the trajectories of myeloid, erythroid, and lymphoid lineages using Microwell-seq (Fig. 6b, Fig. S12D-E and Supplementary Table 13). Interestingly, these subclones exhibited poor correlation with cell phenotypes. All the MT mutation subclones were detected in different cell lineage clusters, suggesting that multiple cell clones might contribute to the AML hierarchy, and different cells might fall into a similar type of “attractor” in AML (Fig. 6c, Fig. S12D, E) [52].

Theoretical studies suggested that complex networks may exhibit ordered or stable dynamics, raising the possibility that the fates of cell may represent attractor states [53]. Based on our observation of shared AML progenitor stages among different patients and shared single-cell transcriptome states among different MT mutation cell clones, we proposed the existence of a “cancer attractor”. To explore the transcriptional regulation network (TRN) of this potential cancer attractor state, we applied an algorithm for the reconstruction of accurate cellular networks (ARACNe) and VIPER to form the network [41, 54]. Fig. 6d-f shows the core genes of the attractor networks of AML progenitor cells (D), AML (E) and normal (F) myeloid cells in comparison to HSPCs in P25 patients. YBX1 and NFE2L2 were detected in all the three networks, while CEBPD, SPI1, GFI1, NCOR1, and ZNF780A were only in normal and AML myeloid cell. CAMTA1, CEBPG, HOXA10, LRRFIP1, and MAFB were shared by AML myeloid and progenitor cell. Other genes in Fig. 6d, such as MYB, RUNX2, and YY1, were unique in AML progenitor cells of P25 and could be the determinants of its cancer attractor. P10 and P17 also had their own attractor networks (Fig. S12F-I).

### AML target searching based on the HCL

Since the HCL has been constructed, it triggered our interests to explore the DEGs, which are high in AML but low in other tissues at single-cell level (Fig. S13A) [25].

**Fig. 7:**
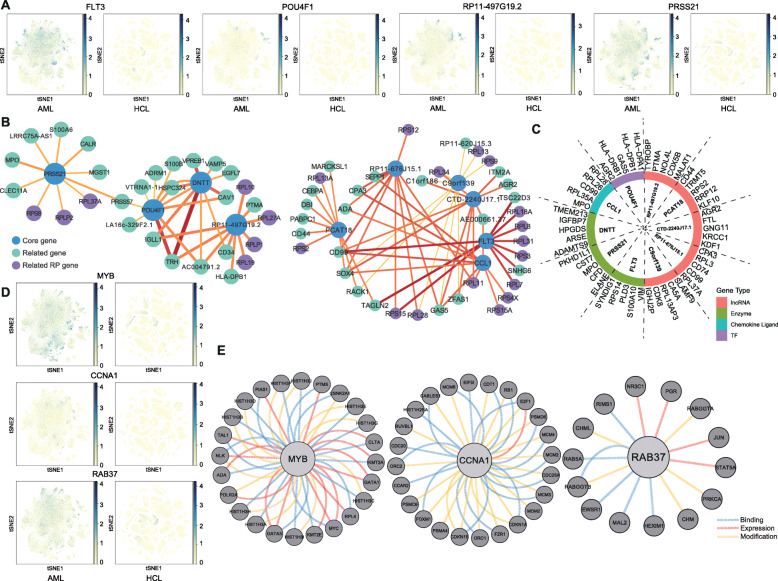
AML target searching based on the HCL. **a** Feature plots of genes in AML map and HCL. The left panel is the AML map and the right is HCL. Gene expression levels are indicated by blue and yellow. **b** Correlation networks of the top 10 highly expressed genes and their most relevant genes in AML. The top expressed genes are the core genes in blue. The most relevant genes are in green and purple (RPs). **c** The top expressed AML genes and their most relevant protein-coding genes in AML progenitor cluster. **d** Feature plots of MYB, CCNA1, and RAB37 in the AML map and the HCL. The left panel is AML map and the right is HCL. Gene expression levels are indicated by blue and yellow. **e** The interacting gene analysis using pathway commons. The blue, red, and yellow lines represent the binding, expression-controlled, and state change-controlled (modification) genes, respectively

After the comparison of two databases, we got a list of highly expressed genes in AML (Supplementary Table 14). Among the top 10 genes, FLT3 is well-known, and POU4F1 has also been reported [55]. Moreover, half of them are lncRNA. Others are PRSS21, CCL1, and DNTT (Fig. 7a and Fig. S13B). Correlation analysis revealed that these top 10 genes belong to two networks along with their most relevant genes in AML (Fig. 7b). The RP genes play important roles in both networks. Further, we investigated the top 5 relevant protein-coding genes in AML progenitor cluster which turned out to be tumor or myeloid differentiation-related (Fig. 7c) [56–61].

Combined with the TCGA, we found that there were three other genes that contributed to the malignancy (Fig. 7d and Fig. S13C). MYB is a novel TF in cancers [62]. Although it could hardly be targeted directly, we could pay attention to the interacting genes. CCNA1, a cyclin, and RAB37, a GTPase, are less discussed. They and their linked genes might also be potential targets (Fig. 7e and Fig. S13D).

Altogether, we believe that the comparison of gene expression atlases between AML and normal HCL will be helpful for the exploration of novel and specific targets.

## Discussion

The emergence of the single-cell technologies permits the dissection of cellular heterogeneity with genome, epigenome, transcriptome, and proteome analyses [63, 64]. Advances in technology deepens our understanding of the molecular mechanism underlying healthy and malignant hematopoiesis [65]. Previous studies have been designed to study leukemia from diagnosis to prognosis [12, 66–68]. However, limited scales and technical consistency constrained them to draw a generalized picture of AML at the single-cell level. Herein using Microwell-seq, a high-throughput single-cell mRNA sequencing platform, we collected data from a large number of cells and carried out an integrative analysis on up to 40 patients.

Previous studies have reported the deregulation of ribosomal proteins (RPs) in human malignancies [15]. RPs confer a selective advantage to malignant cells [16]. They have been associated with malignant cells through extra ribosomal functions related to proliferation, DNA repair, apoptosis, and cellular homeostasis [69]. In addition, they play a critical role in the acquisition and maintenance of cancer stem cell phenotype [70]. The impairment of ribosome biogenesis leads to p53 induction and cell cycle arrest [71]. Innovative drugs, which hinder ribosome biogenesis to stabilize p53, have shown preclinical activity and are currently in early clinical development in hematological malignancies [72]. In our study, we found that the AML progenitor cells were characterized by a high expression level of multiple RP genes, which were involved in the p53 pathway. The dysregulation of transcriptome might lead to failure of remission.

There were limitations and bias in the comparison of the CR rate and survival rate in type I and II patients in our study. Our sample size was small, and we were not able to track all the patients. Some patients chose other hospitals for better treatment, and some patients, especially the elderly ones, go back home without treatment, or died from other diseases at the beginning of treatment, such as cerebral hemorrhage and atrial fibrillation. The elderly patients with good prognosis were more likely to receive continuous treatment, and this might lead to bias.

The combination of the next-generation sequencing and the targeted long-read sequencing is able to identify the mutations at a single-cell level. The targeted sequencing is for bulk sample, ignoring the cluster heterogeneity. Single-cell next-generation sequencing is harped by the coverage. It only sequences 150 bp from poly A, failing to identify mutations, which usually locate thousands of bases away. Previous studies have combined long-read nanopore sequencing with short-read based transcriptome profiling of barcoded single cells to track the clonal changes [73, 74]. Though nanopore sequencing provides high throughput, the SMRT sequencing of PacBio sequences a molecule multiple times to generate high-quality data and has a better overall performance [75]. In our study, SMRT sequencing was combined with Microwell-seq, so that the mutations with barcodes could be detected and associated with cell transcriptome.

Mitochondrial mutations are usually heteroplasmic, and the cell can tolerate a high percentage level of this variant before the biochemical threshold is exceeded [76]. Our study regarded the mtDNA mutation as the clue of lineage tracing and found that the same phenotype contained multiple clones, implying that there were certain key attractors responsible for determining the switching between different states [77].

As Waddington’s landscape has explained, the attractor state is regulated by underlying gene regulatory network [78]. Based on theory of gene regulatory networks, cancer cells also represent attractor states of the network dynamics [79]. Our study described the gene regulatory networks of AML progenitor cells and made comparisons with the normal and AML myeloid cell. For therapeutic purposes, it gives us a hint that drugs which help tumor cells exit from the cancer attractor and entry into a benign attractor may reduce tumor burden [80].

The treatment of AML has changed substantially in recent years. New targeted drugs have emerged, including midostaurin and gilteritinib to target FLT3, and ivosidenib and enasidenib to target mutant isocitrate dehydrogenase 1 and 2 [81]. The best responses to treatment are seen when these agents are combined with conventional chemotherapy [82]. Based on the comparison between AML map and HCL at a single-cell level, we proposed CCNA1 and RAB37 as new potential drug targets. They are highly expressed in only AML progenitor cell cluster rather than other tissues. Cell cycle regulators are considered attractive targets in cancer therapy [83]. CCNA1 is a suitable immuno-therapeutic target for future clinical trials, and generating donor-derived CCNA1-specific T cells seems to be a possible approach to prolonged disease remission in post-HSCT patients [84]. An aberrant expression of Rab proteins has been reported in multiple cancer types [85]. The underlying mechanism of RAB37 in lung cancer has been widely discussed [86]. However, there has been no study in AML. Moreover, transcription factors like MYB and some lncRNAs have significantly different levels of expression between AML progenitor cells and normal tissues. Even though they have barely been considered as priority targets, focusing on their interacting proteins might control their expressions [62, 87]. We hope that our study will bring new insights into AML targeted therapy.

## Conclusions

In our study, we analyzed 191727 cells from 40 patients with AML using Microwell-seq to establish a single-cell AML landscape. We identified a malignant AML progenitor cell cluster with novel AML markers. Patients with RP high progenitor cells had a low remission rate. Based on the AML landscape, we deduced two types of AML with diverse clinical outcomes. We showed that a single-cell analysis could be used to predict and evaluate the therapeutic effect. Finally, by combining Microwell-seq with SMRT sequencing, we traced mitochondrial mutations in the AML landscape and demonstrated a lack of association between AML clones and the transcriptomic phenotypes. Our results suggest that the existence of a phenotypic “cancer attractor” might help to define a common phenotype for AML progenitor cells.

## Methods

### Patient samples and single-cell preparation

Samples were obtained from newly diagnosed patients with AML at the 1st Affiliated Hospital of Zhejiang University. Patients with AML were diagnosed according to the FAB classification. Patients diagnosed with other leukemia types were excluded. Patients with no clinical symptoms with blast cells in BM < 5%, hemoglobin concentration > 90 g/L, platelet > 100 × 10^9^/L, and normal white blood cells counts were considered to be in CR. The patients were considered to be in partial remission when the blast cells in BM < 20%, but > 5%. For relapse, the blast cells in BM > 20% again after remission. In our study, patients who were not in remission or partial remission after the second regimen were considered to be refractory patients. Cells were isolated from bone marrow aspirates by Ficoll Hypaque Solution (Haoyang Institute of Biotechnology, Tianjin, China), and diluted to ≈ 200000/ml for Microwell-seq in DPBS.

### Microwell-seq

After fabricating the microwell device and synthesizing the barcoded beads, cells and beads were pipetted onto the microwell array for lysis. Through reverse transcription, exonuclease I treatment, cDNA amplification, transposase fragmentation, and selective PCR, the samples were ready for sequencing on Illumina Hiseq system finally. For the protocol of Microwell-seq, please refer to our previous articles [21].

### Processing of the Microwell-seq data

Standard procedures for processing the Microwell-seq datasets were performed using the protocols described in the previously published paper [21]. Reads were aligned to the Homo_sapiens GRCh38 genome. For cell quality control, we only retained cells in which more than 500 transcripts were expressed. Moreover, cells with high proportion of transcript counts derived from mitochondria-encoded genes were also excluded. We used Seurat to perform clustering analysis of single-cell data from different patients [88, 89]. The filtered digital gene expression data was log2 (TPM/100 + 1) transformed, and the number of UMI and the percentage of mitochondrial gene content were regressed out. We calculated around 2000 genes that exhibit the highest cell-to-cell variation in the dataset for initial principal component analysis (PCA). Subsequently, we performed nonlinear dimensional reduction (t-SNE) analysis with the presumed number of PCA by the PCElbowPlot function and JackStrawPlot function. Next, we used the FindCluster function for clustering cells and applied default Wilcoxon rank sum test to find markers expressed differently in each cluster.

Dealing with the huge datasets, we performed a linear regression on all genes to eliminate batch effects (ScaleData function) and drew the 40 AMLs map using the Python-based package Scanpy [90]. Taking into account the gene expression in malignant cells largely set by patient-specific factors, in order to explore the heterogeneity of AML progenitor cells, we first worked to remove features from a single patient. We first clustered the malignant cells of each patient into many small subgroups without supervision at a high resolution and calculated the average gene expression of the cells in each subgroup as its expression characteristic. These subgroups were clustered into different modules using the method of ward. We discarded D2 and those consisting of only a single patient. Then, the main signature genes of each remaining module were found using the function FindAllMarkers in Seurat. We subsequently used the 500 more signature genes found to cluster all AML progenitor single cells in Seurat according to the above process. To reveal the heterogeneity in refractory and non-refractory patients, we randomly selected 12000 cells in refractory and non-refractory patients. Seurat3 was used to integrate different datasets for comparative analysis (https://satijalab.org/seurat/v3.0).

### PAGA analysis of cell clustering

We constructed a symmetrized kNN-like graph in order to reveal the relationships among PBMC, BMMC, and HSPC, using the approximate nearest neighbor search with ForceAtlas2. We adopted the Louvain algorithm in the implementation at suitable resolutions to determine all partitionings of interest of the kNN-like graph [91].

### Single-cell trajectory analysis

Using PAGA analysis, we estimated pseudotime. An extended version of diffusion pseudotime reference that accounted for disconnected graphs was used. It consisted in a simple modification of the original algorithm that accounted for disconnected Eigen-subspaces of the graph adjacency matrix, which resulted in multiple subspaces of Eigen value 1 of the graph transition matrix. We assigned an infinite distance to cells that resided in disconnected clusters and computed distances among cells within connected regions in the graph. For PAGA path, it averaged all single-cell paths that passed through the corresponding groups of cells and permitted the tracing of gene expression changes along complex trajectories at single-cell resolution [91].

### Cell-cell interaction network

We built cell-cell correlation-based networks in order to understand the relationships between different subgroups. The gene expression profiles for the AML map were normalized to the total number of transcripts and multiplied by 100000. We equally used pseudo-cells. Each pseudo-cell was an average of 50 cells with most genes detected from the same cell type. Using Pearson’s correlation, we formed a correlation network between these cells. Edges with *r* > 0.65 were considered significant. The network was visualized using Cytoscape with the “edge-weighted spring embedded” layout [92].

### Correlation analysis

The gene expression profiles for the AML map were normalized to the total number of transcripts and multiplied by 100000. We used pseudo-cells to reduce the effects of noise and outliers. Each pseudo-cell was an average of 20 cells randomly selected from the same cell type [93]. Then in order to compare the relationships of each cell type, the MetaNeighbor analysis was performed [94].

### Single-cell blast analysis

In our previous study, we employed the scHCL reference and the normal bone marrow reference, which was built in the same method of integrating the top 20 marker genes for each normal bone marrow cluster to a gene list and used the average expression values. Pearson’s correlations between the sample cells and cell types were calculated, and the highly correlated relationship were shown by the heatmap.

### Differential expression analysis

Differential expression tests were performed using MAST, which fits a hurdle model to the expression of each gene, consisting of logistic regression for the zero process (i.e., whether the gene is expressed) and linear regression for the continuous process (i.e., the expression level) [95]. In order to understand the characteristics of the AML progenitor cell cluster, we first compared AML progenitor cells and HSPCs to control myeloid cells respectively, then compared each against the remaining two to obtain cell type-specific genes. Specifically, we used the regression formula “Expi = nGene + Celltype”, where “Expi” is the standardized log2 (TP10K + 1) expression vector for gene i across all cells, “Celltype” is a binary variable reflecting cell identity, and “nGene” is the number of genes detected in each cell. Cells were evenly downsampled across groups so that a maximum of 10000 cells were tested for each cell type. The discrete and continuous coefficients of the model were both retrieved and *p* values were calculated using the likelihood ratio test in MAST. Differential expression coefficients and *p* values corresponding to the discrete component were used for the heatmap and volcano plot and subsequent analyses.

### Gene expression difference analysis with TCGA high-throughput (HT) sequencing data

We downloaded HT seq count datasets with 274 samples of “Primary Blood Derived Cancer−Bone Marrow” in TCGA and 4 samples of “Healthy Bone Marrow” in GSE118476 for gene expression difference analysis. The R package DEseq2 was used to normalize the HT seq count datasets; then, the R package ggplot2 and ggpubr were used to perform Wilcoxon’s test and to add significant difference indicators.

### Gene co-expression network analysis

The gene expression profiles for AML progenitor cells were normalized to the total number of transcripts and multiplied by 100000. We then used pseudo-cells for subsequent analyses. Each pseudo-cell was an average of 20 cells randomly selected from the same AML progenitor cell sub-cluster. Thereafter, we inputed the expression matrix of these pseudo-cells into WGCNA to calculate the gene co-expression network in tumor cells. In order to acquire hub genes inside the module, we calculated Module Membership and its *p* value for each gene in the module, and selected genes with a module membership > 0.6 and a *p* value < 0.01. Then, we exported the topological overlap matrix of these hub genes to Cytoscape. Using Cytoscape, we visualized the network with the “edge-weighted spring embedded” layout.

### Pre and post regimen comparison

We performed MetaNeighbor analysis in order to compare the clusters pre- and post-treatment, and got the area under the receiver operator characteristic curve (AUROC) scores between cell types in different batches based on the highly variable genes. We used 0.7 as the AUROC score threshold to show the similarity. We used the Circlize package to view the similarity of cell types [96].

### GSEA

GO Enrichment analysis of AML progenitor cell marker genes was performed and presented using an online tool called Metascape (http://metascape.org). Only the top 100 marker genes of AML progenitor cells and top 50 marker genes of AML progenitor sub-clusters with the highest Wilcoxon test scores were chosen. The enrichment of refractory signatures of C5 on the C13 signatures was performed by GSEA. The GSEA was implemented using JAVA downloaded from the Broad Institute (http://software.broadinstitute.org/gsea). The master regulator analysis was also performed by comparing C5 against C13 using the msviper algorithm of the VIPER package (http://bioconductor.org/packages/release/bioc/html/viper.html).

### Survival analysis

The survival analysis of MYC, SRC, RELA, and MTOR was conducted by using the c-BioPortal (http://www.cbioportal.org) [97, 98]. The log-rank test was used to analyze the overall survival rate of 200 AML samples from TCGA. The samples were divided into subgroups by mRNA expression *Z*-scores of targeted genes and the *p* value was automatically calculated according to the online instructions. The log-rank test was also used to analyze survival rate of type I and II patients.

### Long-read single-cell targeted SMRT sequencing

The upstream primer of MT-ND4 was ATGCTAAAACTAATCGTCCCA. The universal downstream primer was TGGTATCAACGCAGAGTAC-s-G-s-T. We used SMRT Portal, an analytical platform based on a small server in the lab, after the amplification and sequencing, to get circular consensus sequencing (CCS) of which quality scores were above 0.9. Then, we used three linker sequences to further filter the reads and got the reads including the section that matched the sequence “ACGT******CGACTCACTACAGGG (linker1 sequence) ******TCGGTGACACGATCG (linker2 sequence) ************ TTTTTTTTTTTT (linker3 sequence)” for further analysis. Acquired reads were aligned to transcript references that consisted only of the targeted gene using minimap2 by the following parameters: -ax splice -u b -C5 -N0. Only the well-mapped reads were reserved. Mutations were called using GATK Mutect2 (version 3.8-1, https://github.com/broadgsa/gatk-protected.git) and visualized by IGV. Then, we projected different variants onto the UMAP based on the corresponding cell barcode. We quantified the intersections of mitochondria mutations by the Upset R package for mitochondria lineage inference. The intersections of mitochondria mutations showed that cells were separated by several sets of different variants [20].

### Master regulator analysis

We deduced the TFs that were candidate drivers of different cell states using the msviper algorithm of the VIPER package (http://bioconductor.org/packages/release/bioc/html/viper.html). We first used the ARACNe to deduce the regulon associated with transcription factors in paired gene expression signatures for normal myeloid cells and AML progenitor cells against HSPCs (https://github.com/califano-lab/ARACNe-AP). The msviper algorithm prioritized the transcription factors that were the most likely determinants of an observed differential expression signature related to state transition. Most obviously enriched VIPER-inferred transcription factors for cell states in normal myeloid and AML progenitor cells against HSPC were chosen to plot the viper heatmap. For attractor network inference, we noticed that the downstream targets of each deduced transcription factor identified by ARACNe might not be expressed differently. Therefore, we pruned it by removing non-differentially expressed downstream targets (FDR > 0.05). Subsequently, for each attractor state, we selected transcription factors containing the DEGs that were the most highly differentially expressed. We initially calculated a gene score for each downstream target by multiplying absolute log2(fold change) and − log10(FDR). For the most obviously enriched VIPER-inferred TFs (FDR < 0.05 and NES > 0), we removed a TF from our analysis if its target gene scores were not significantly higher than the gene scores of all the other DEGs via a one-sided Wilcoxon’s non-parametric test. This ensured that the remaining TFs had downstream targets with gene scores significantly higher than the gene scores of the DEGs that were not part of each respective TF. Therefore, the remaining TFs had downstream targets representing the most highly DEGs. Then, we drew an attractor network using the selected TFs whose Pearson’s correlation coefficients were calculated using the above ARACNe-inferred regulating relationship. Edges with *r* > 0.3 were considered significant. The network was visualized using Cytoscape with the “edge-weighted spring embedded” layout.

### Comparison analysis with the HCL

We performed pseudo-cell processing on the AML progenitor cells and HCL data. Each pseudo-cell was an average of 20 cells randomly selected from the same cell cluster. HCL data randomly sampled 50 cells per cluster (clusters with less than 50 cells retained the original cells). Then, we performed standardized CPM processing and log1p processing. We used the FindMarkers function and FindAllMarkers function in the R package Seurat and analyze the DEGs of 102 HCL cell clusters (Parameters: min.pct = 0.25, min.diff.pct = 0.25, logfc.threshold = 0.25). We extracted the top 20 marker genes from each HCL cluster and filtered out this part of genes in the gene set specifically expressed in AML progenitor cells. Finally, we used the sc.pl.tsne function in Scanpy to present the overall expression of specific genes in AML and HCL.

### Gene-gene correlation analysis and network

After the pseudo-cell processing above and filtering the genes expressed in < 3 cells in AML dataset, we used the Python package pandas to calculate the Pearson’s correlation coefficients between genes. After the absolute-value processing, we constructed a correlation matrix. We used the “Circular Layout” in Cytoscape to visualize the gene-gene correlation network in the top 10 DEGs and the genes with the highest correlation coefficients in AML BMMCs. The R package ggplot2 was used to present the related top 5 protein-coding genes in AML progenitor cluster.

Gene interaction network was conducted using the Pathway Commons (http://www.pathwaycommons.org) and Cytoscape. The TRN was conducted using the Pathway Net (http://pathwaynet.princeton.edu). The minimum relationship confidence was 0.5 and the maximum number of genes was 20.

## Supplementary information

**Additional file 1: Fig. S1.** Workflow and Individual t-SNE maps of normal donors.

**Additional file 2: Fig. S2.** Neutrophil clusters and the marker genes.

**Additional file 3: Fig. S3.** Individual t-SNE maps of P01-P20.

**Additional file 4: Fig. S4.** Individual t-SNE maps of P21-P40.

**Additional file 5: Fig. S5.** Single-cell blast analysis.

**Additional file 6: Fig. S6.** Gene expression levels in TCGA.

**Additional file 7: Fig. S7.** Metascape enrichment network, VIPER and FLT3 analyses.

**Additional file 8: Fig. S8.** Intratumoral heterogeneity in AML progenitor cells.

**Additional file 9: Fig. S9.** Individual t-SNE maps of two extra patients and two patients post regimens.

**Additional file 10: Fig. S10.** Clinical implication of P-extra 2.

**Additional file 11: Fig. S11.** Intratumoral heterogeneity in monocytic leukemia.

**Additional file 12: Fig. S12.** Genetic mutation revealed by SMRT sequencing.

**Additional file 13: Fig. S13.** Target searching based on the HCL.

**Additional file 14: Table S1**. Marker genes of individual t-SNE map

**Additional file 15: Table S2.** Marker genes of normal BMMCs and neutrophils.

**Additional file 16: Table S3.** Marker genes of PAGA analysis. **Table S4.** Clinical characteristics. **Table S5.** Marker genes of AML BMMCs. **Table S6.** Common and unique genes in HSPCs and AML progenitor cells vs. myeloid cells

**Additional file 17: Table S7.** Marker genes in subdivision t-SNE map of AML progenitor cell cluster. **Table S8** DEGs in RP gene high clusters. **Table S10.** The up and downregulated genes in P20-Post in comparison to P20-Pre. **Table S11**. Marker genes of refractory and non-refractory cells. **Table S14.** Highly expressed genes in AML in comparison to HCL.

**Additional file 18: Table S9.** Marker genes in UMAP pre and post regimen.

**Additional file 19: Table S12.** GSEA of C5 in comparison to C13.

**Additional file 20: Table S13.** Marker genes of P25, P10, and P17 in Monocle3-UMAP.

## Data Availability

scRNA-seq data have been deposited in NCBI GEO with accession GSE130756.

## Abbreviations

AML: Acute myeloid leukemia
RPs: Ribosomal proteins
BMMC: Bone marrow mononuclear cell
t-SNE: t-Distributed stochastic neighbor embedding
HSPC: Hemopoietic stem/progenitor cell
PBMC: Peripheral blood mononuclear cell
PAGA: Patition-based graph abstraction
DEGs: Differentially expressed genes
TF: Transcription factor
VIPER: Virtual inference of protein-activity by enriched regulon
WGCNA: Weighted correlation network analysis
CR: Complete remission
UMAP: Uniform approximation and projection
GSEA: Gene set enrichment analysis
HCL: Human Cell Landscape
TRN: Transcriptional regulation network
ARACNe: Algorithm for the reconstruction of accurate cellular networks
PCA: Principal component analysis
AUROC: Area under the receiver operator characteristic curve
CCS: Circular consensus sequencing

## References

[1] Dohner H, Estey E, Grimwade D, Amadori S, Appelbaum FR, Buchner T (2017). Diagnosis and management of AML in adults: 2017 ELN recommendations from an international expert panel. Blood..

[2] Bullinger L, Dohner K, Dohner H (2017). Genomics of acute myeloid leukemia diagnosis and pathways. J Clin Oncol.

[3] Luppi M, Fabbiano F, Visani G, Martinelli G, Venditti A (2018). Novel agents for acute myeloid leukemia. Cancers (Basel).

[4] Timilshina N, Breunis H, Tomlinson GA, Brandwein JM, Buckstein R, Durbano S (2019). Long-term recovery of quality of life and physical function over three years in adult survivors of acute myeloid leukemia after intensive chemotherapy. Leukemia..

[5] Grimwade D, Ivey A, Huntly BJP (2016). Molecular landscape of acute myeloid leukemia in younger adults and its clinical relevance. Blood..

[6] Klco JM, Spencer DH, Miller CA, Griffith M, Lamprecht TL, O'Laughlin M (2014). Functional heterogeneity of genetically defined subclones in acute myeloid leukemia. Cancer Cell.

[7] Tamamyan G, Kadia T, Ravandi F, Borthakur G, Cortes J, Jabbour E (2017). Frontline treatment of acute myeloid leukemia in adults. Crit Rev Oncol Hematol.

[8] Zeijlemaker W, Grob T, Meijer R, Hanekamp D, Kelder A, Carbaat-Ham JC, et al. CD34(+)CD38(-) leukemic stem cell frequency to predict outcome in acute myeloid leukemia. Leukemia. 2018. 10.1038/s41375-018-0326-3 30542144

[9] Wang Y, Navin NE (2015). Advances and applications of single-cell sequencing technologies. Mol Cell.

[10] Chen H, Ye F, Guo G (2019). Revolutionizing immunology with single-cell RNA sequencing. Cell Mol Immunol.

[11] Povinelli BJ, Rodriguez-Meira A, Mead AJ. Single cell analysis of normal and leukemic hematopoiesis. Mol Aspects Med. 2017. 10.1016/j.mam.2017.08.006 PMC5771467 28863981

[12] Pellegrino M, Sciambi A, Treusch S, Durruthy-Durruthy R, Gokhale K, Jacob J (2018). High-throughput single-cell DNA sequencing of acute myeloid leukemia tumors with droplet microfluidics. Genome Res.

[13] Paguirigan AL, Smith J, Meshinchi S, Carroll M, Maley C, Radich JP (2015). Single-cell genotyping demonstrates complex clonal diversity in acute myeloid leukemia. Sci Transl Med.

[14] Hanahan D, Weinberg RA (2011). Hallmarks of cancer: the next generation. Cell..

[15] Shenoy N, Kessel R, Bhagat TD, Bhattacharyya S, Yu Y, McMahon C (2012). Alterations in the ribosomal machinery in cancer and hematologic disorders. J Hematol Oncol.

[16] Guimaraes JC, Zavolan M (2016). Patterns of ribosomal protein expression specify normal and malignant human cells. Genome Biol.

[17] Khajuria RK, Munschauer M, Ulirsch JC, Fiorini C, Ludwig LS, McFarland SK (2018). Ribosome levels selectively regulate translation and lineage commitment in human hematopoiesis. Cell..

[18] Liu JM, Ellis SR (2006). Ribosomes and marrow failure: coincidental association or molecular paradigm?. Blood..

[19] Ludwig LS, Lareau CA, Ulirsch JC, Christian E, Muus C, Li LH (2019). Lineage tracing in humans enabled by mitochondrial mutations and single-cell genomics. Cell..

[20] Xu J, Nuno K, Litzenburger UM, Qi Y, Corces MR, Majeti R, et al. Single-cell lineage tracing by endogenous mutations enriched in transposase accessible mitochondrial DNA. Elife. 2019;8. 10.7554/eLife.45105 PMC6469926 30958261

[21] Han X, Wang R, Zhou Y, Fei L, Sun H, Lai S (2018). Mapping the mouse cell atlas by microwell-seq. Cell..

[22] Velten L, Haas SF, Raffel S, Blaszkiewicz S, Islam S, Hennig BP (2017). Human haematopoietic stem cell lineage commitment is a continuous process. Nat Cell Biol.

[23] Azizi E, Carr AJ, Plitas G, Cornish AE, Konopacki C, Prabhakaran S (2018). Single-cell map of diverse immune phenotypes in the breast tumor microenvironment. Cell..

[24] Lai S, Huang W, Xu Y, Jiang M, Chen H, Cheng C (2018). Comparative transcriptomic analysis of hematopoietic system between human and mouse by Microwell-seq. Cell Discov.

[25] Han X, Zhou Z, Fei L, Sun H, Wang R, Chen Y, et al. Construction of a human cell landscape at single-cell level. Nature. 2020. 10.1038/s41586-020-2157-4 32214235

[26] Vodyanik MA, Thomson JA, Slukvin II (2006). Leukosialin (CD43) defines hematopoietic progenitors in human embryonic stem cell differentiation cultures. Blood..

[27] Novershtern N, Subramanian A, Lawton LN, Mak RH, Haining WN, McConkey ME (2011). Densely interconnected transcriptional circuits control cell states in human hematopoiesis. Cell..

[28] Okada S, Fukuda T, Inada K, Tokuhisa T (1999). Prolonged expression of c-fos suppresses cell cycle entry of dormant hematopoietic stem cells. Blood..

[29] Jan T, Pittois K, NicolaI P, Joseph M, Angel P (2000). Collagenase-3 (MMP-13) and integral membrane protein 2a (Itm2a) are marker genes of chondrogenic/osteoblastic cells in bone formation: sequential temporal, and spatial expression of Itm2a, alkaline phosphatase, MMP-13, and osteocalcin in the mouse. J Bone Miner Res.

[30] Bertoli S, Paubelle E, Berard E, Saland E, Thomas X, Tavitian S (2019). Ferritin heavy/light chain (FTH1/FTL) expression, serum ferritin levels, and their functional as well as prognostic roles in acute myeloid leukemia. Eur J Haematol.

[31] Laverdiere I, Boileau M, Herold T, Rak J, Berdel WE, Wormann B (2016). Complement cascade gene expression defines novel prognostic subgroups of acute myeloid leukemia. Exp Hematol.

[32] Bertrand J, Despeaux M, Joly S, Bourogaa E, Gallay N, Demur C (2012). Sex differences in the GSK3beta-mediated survival of adherent leukemic progenitors. Oncogene..

[33] Melillo L, Cascavilla N, Lombardi G, Carotenuto M (1992). P M. Prognostic relevance of serum beta 2-microglobulin in acute myeloid leukemia. Leukemia..

[34] Bertazzoni U, Brusamolino E, Isernia P, Scovassi AI, Torsello S, Lazzarino M (1982). Prognostic significance of terminal transferase and adenosine deaminase in acute and chronic myeloid leukemia. Blood..

[35] Vaikari VP, Du Y, Wu S, Zhang T, Metzeler K, Batcha AMN, et al. Clinical and preclinical characterization of CD99 isoforms in acute myeloid leukemia. Haematologica. 2019. 10.3324/haematol.2018.207001 PMC7109747 31371417

[36] Liu L, Luo C, Luo Y, Chen L, Liu Y, Wang Y (2018). MRPL33 and its splicing regulator hnRNPK are required for mitochondria function and implicated in tumor progression. Oncogene..

[37] Chiang CY, Pan CC, Chang HY, Lai MD, Tzai TS, Tsai YS (2015). SH3BGRL3 protein as a potential prognostic biomarker for urothelial carcinoma: a novel binding partner of epidermal growth factor receptor. Clin Cancer Res.

[38] Bouchal P, Dvorakova M, Roumeliotis T, Bortlícek Z, Ihnatova I, Prochazkova I (2015). Combined proteomics and transcriptomics identifies carboxypeptidase B1 and nuclear factor B (NF- B) associated proteins as putative biomarkers of metastasis in low grade breast cancer. Mol Cell Proteomics.

[39] Bjorkblom B, Padzik A, Mohammad H, Westerlund N, Komulainen E, Hollos P (2012). c-Jun N-terminal kinase phosphorylation of MARCKSL1 determines actin stability and migration in neurons and in cancer cells. Mol Cell Biol.

[40] Scotlandi K, Remondini D, Castellani G, Manara MC, Nardi F, Cantiani L (2009). Overcoming resistance to conventional drugs in Ewing sarcoma and identification of molecular predictors of outcome. J Clin Oncol.

[41] Alvarez MJ, Shen Y, Giorgi FM, Lachmann A, Ding BB, Ye BH (2016). Functional characterization of somatic mutations in cancer using network-based inference of protein activity. Nat Genet.

[42] Shahrin NH, Diakiw S, Dent LA, Brown AL, D'Andrea RJ (2016). Conditional knockout mice demonstrate function of Klf5 as a myeloid transcription factor. Blood..

[43] Kawaida R, Ohtsuka T, Okutsu J, Takahashi T, Kadono Y, Oda H (2003). Jun dimerization protein 2 (JDP2), a member of the AP-1 family of transcription factor, mediates osteoclast differentiation induced by RANKL. J Exp Med.

[44] Chatterjee SS, Biswas M, Boila LD, Banerjee D, Sengupta A (2018). SMARCB1 deficiency integrates epigenetic signals to oncogenic gene expression program maintenance in human acute myeloid leukemia. Mol Cancer Res.

[45] Sun Y, Zhou B, Mao F, Xu J, Miao H, Zou Z (2018). HOXA9 reprograms the enhancer landscape to promote leukemogenesis. Cancer Cell.

[46] Roche J, Zeng C, Baron A, Gadgil S, Gemmill RM, Tigaud I (2004). Hox expression in AML identifies a distinct subset of patients with intermediate cytogenetics. Leukemia..

[47] Ozeki K, Kiyoi H, Hirose Y, Iwai M, Ninomiya M, Kodera Y (2004). Biologic and clinical significance of the FLT3 transcript level in acute myeloid leukemia. Blood..

[48] Walter RB, Othus M, Burnett AK, Lowenberg B, Kantarjian HM, Ossenkoppele GJ (2013). Significance of FAB subclassification of “acute myeloid leukemia, NOS” in the 2008 WHO classification: analysis of 5848 newly diagnosed patients. Blood..

[49] Arber DA, Orazi A, Hasserjian R, Thiele J, Borowitz MJ, Le Beau MM (2016). The 2016 revision to the World Health Organization classification of myeloid neoplasms and acute leukemia. Blood..

[50] Thol F, Schlenk RF, Heuser M, Ganser A (2015). How I treat refractory and early relapsed acute myeloid leukemia. Blood..

[51] Wallace DC, Chalkia D (2013). Mitochondrial DNA genetics and the heteroplasmy conundrum in evolution and disease. Cold Spring Harb Perspect Biol.

[52] Mojtahedi M, Skupin A, Zhou J, Castano IG, Leong-Quong RY, Chang H (2016). Cell fate decision as high-dimensional critical state transition. PLoS Biol.

[53] Huang S, Eichler G, Bar-Yam Y, Ingber DE (2005). Cell fates as high-dimensional attractor states of a complex gene regulatory network. Phys Rev Lett.

[54] Lachmann A, Giorgi FM, Lopez G, Califanoy A (2016). ARACNe-AP: gene network reverse engineering through adaptive partitioning inference of mutual information. Bioinformatics..

[55] Fortier JM, Payton JE, Cahan P, Ley TJ, Walter MJ, Graubert TA (2010). POU4F1 is associated with t(8;21) acute myeloid leukemia and contributes directly to its unique transcriptional signature. Leukemia..

[56] Tomasello E, Vivier E (2005). KARAP/DAP12/TYROBP: three names and a multiplicity of biological functions. Eur J Immunol.

[57] Zoller M (2015). CD44, hyaluronan, the hematopoietic stem cell, and leukemia-initiating cells. Front Immunol.

[58] Delom F, Nazaraliyev A, Fessart D (2018). The role of protein disulphide isomerase AGR2 in the tumour niche. Biol Cell.

[59] Lo PHY, Lung HL, Cheung AKL, Apte SS, Chan KW, Kwong FM (2010). Extracellular protease ADAMTS9 suppresses esophageal and nasopharyngeal carcinoma tumor formation by inhibiting angiogenesis. Cancer Res.

[60] HAZNEDAROGLU IC, MALKAN UY (2016). Local bone marrow renin-angiotensin system in the genesis of leukemia and other malignancies. Eur Rev Med Pharmacol Sci.

[61] Dollt C, Michel J, Kloss L, Melchers S, Schledzewski K, Becker K (2018). The novel immunoglobulin super family receptor SLAMF9 identified in TAM of murine and human melanoma influences pro-inflammatory cytokine secretion and migration. Cell Death Dis.

[62] Mitra P (2018). Transcription regulation of MYB: a potential and novel therapeutic target in cancer. Ann Transl Med.

[63] Hedlund E, Deng Q (2018). Single-cell RNA sequencing: technical advancements and biological applications. Mol Asp Med.

[64] Ye F, Huang W, Guo G (2017). Studying hematopoiesis using single-cell technologies. J Hematol Oncol.

[65] Wang L, Livak KJ, Wu CJ (2018). High-dimension single-cell analysis applied to cancer. Mol Asp Med.

[66] Smith CC, Paguirigan A, Jeschke GR, Lin KC, Massi E, Tarver T (2017). Heterogeneous resistance to quizartinib in acute myeloid leukemia revealed by single-cell analysis. Blood..

[67] Warfvinge R, Geironson L, Sommarin MNE, Lang S, Karlsson C (2017). Single-cell molecular analysis defines therapy response and immunophenotype of stem cell subpopulations in CML. Blood..

[68] Good Z, Sarno J, Jager A, Samusik N, Aghaeepour N, Simonds EF, et al. Single-cell developmental classification of B cell precursor acute lymphoblastic leukemia at diagnosis reveals predictors of relapse. Nat Med. 2018. 10.1038/nm.4505 PMC5953207 29505032

[69] de Las H-RA, Perucho L, Paciucci R, Vilardell J, LL ME (2014). Ribosomal proteins as novel players in tumorigenesis. Cancer Metastasis Rev.

[70] Bastide A, David A (2018). The ribosome, (slow) beating heart of cancer (stem) cell. Oncogenesis..

[71] Fumagalli S, Ivanenkov VV, Teng T, Thomas G (2012). Suprainduction of p53 by disruption of 40S and 60S ribosome biogenesis leads to the activation of a novel G2/M checkpoint. Genes Dev.

[72] Derenzini E, Rossi A, Trere D (2018). Treating hematological malignancies with drugs inhibiting ribosome biogenesis: when and why. J Hematol Oncol.

[73] van Galen P, Hovestadt V, Wadsworth Ii MH, Hughes TK, Griffin GK, Battaglia S (2019). Single-cell RNA-seq reveals AML hierarchies relevant to disease progression and immunity. Cell..

[74] Singh M, Al-Eryani G, Carswell S, Ferguson JM, Blackburn J, Barton K (2019). High-throughput targeted long-read single cell sequencing reveals the clonal and transcriptional landscape of lymphocytes. Nat Commun.

[75] Weirather JL, de Cesare M, Wang Y, Piazza P, Sebastiano V, Wang XJ (2017). Comprehensive comparison of pacific biosciences and oxford nanopore technologies and their applications to transcriptome analysis. F1000Res.

[76] Stewart JB, Chinnery PF (2015). The dynamics of mitochondrial DNA heteroplasmy: implications for human health and disease. Nat Rev Genet.

[77] Li C, Wang J (2015). Quantifying the landscape for development and cancer from a core cancer stem cell circuit. Cancer Res.

[78] CH W (1957). The strategy of the genes.

[79] Lia Q, Wennborga A, Aurellb E, Dekelc E, Zoua J-Z, Xud Y (2016). Dynamics inside the cancer cell attractor reveal cell heterogeneity, limits of stability, and escape. PNAS..

[80] Huang S, Kauffman S (2013). How to escape the cancer attractor: rationale and limitations of multi-target drugs. Semin Cancer Biol.

[81] DiNardo CD, Wei AH (2020). How I treat acute myeloid leukemia in the era of new drugs. Blood..

[82] Stein EM, Tallman MS (2016). Emerging therapeutic drugs for AML. Blood..

[83] Otto T, Sicinski P (2017). Cell cycle proteins as promising targets in cancer therapy. Nat Rev Cancer.

[84] Leung WK, Workineh A, Mukhi S, Tzannou I, Brenner D, Watanabe N (2020). Evaluation of cyclin A1-specific T cells as a potential treatment for acute myeloid leukemia. Blood Adv.

[85] Gopal Krishnan PD, Golden E, Woodward EA, Pavlos NJ, Blancafort P (2020). Rab GTPases: emerging oncogenes and tumor suppressive regulators for the editing of survival pathways in cancer. Cancers (Basel).

[86] Cho SH, Kuo IY, Lu PF, Tzeng HT, Lai WW, Su WC (2018). Rab37 mediates exocytosis of secreted frizzled-related protein 1 to inhibit Wnt signaling and thus suppress lung cancer stemness. Cell Death Dis.

[87] Zimta AA, Tomuleasa C, Sahnoune I, Calin GA, Berindan-Neagoe I (2019). Long non-coding RNAs in myeloid malignancies. Front Oncol.

[88] Satija R, Farrell JA, Gennert D, Schier AF, Regev A (2015). Spatial reconstruction of single-cell gene expression data. Nat Biotechnol.

[89] Butler A, Hoffman P, Smibert P, Papalexi E, Satija R (2018). Integrating single-cell transcriptomic data across different conditions, technologies, and species. Nat Biotechnol.

[90] Wolf FA, Angerer P, Theis FJ (2018). SCANPY: large-scale single-cell gene expression data analysis. Genome Biol.

[91] Wolf FA, Hamey FK, Plass M, Solana J, Dahlin JS, Gottgens B (2019). PAGA: graph abstraction reconciles clustering with trajectory inference through a topology preserving map of single cells. Genome Biol.

[92] Shannon P, Ramage D, Markie A, Amin N (2003). Cytoscape: a software environment for integrated models of biomolecular interaction networks. Genome Res.

[93] Tosches MA, Yamawaki rM, Naumann RK, Jacobi AA, Tushev G, Laurent G (2018). Evolution of pallium, hippocampus, and cortical cell types revealed by single-cell transcriptomics in reptiles. Science.

[94] Crow M, Paul A, Ballouz S, Huang ZJ, Gillis J (2018). Characterizing the replicability of cell types defined by single cell RNA-sequencing data using MetaNeighbor. Nat Commun.

[95] Finak G, McDavid A, Yajima M, Deng J, Gersuk V, Shalek AK (2015). MAST: a flexible statistical framework for assessing transcriptional changes and characterizing heterogeneity in single-cell RNA sequencing data. Genome Biol.

[96] Gu Z, Gu L, Eils R, Schlesner M, Brors B (2014). Circlize implements and enhances circular visualization in R. Bioinformatics..

[97] Cerami E, Gao J, Dogrusoz U, Gross BE, Sumer SO, Aksoy BA (2012). The cBio cancer genomics portal: an open platform for exploring multidimensional cancer genomics data. Cancer Discov.

[98] Gao J, Aksoy BA, Dogrusoz U, Dresdner G, Gross B, Sumer SO (2013). Integrative analysis of complex cancer genomics and clinical profiles using the cBioPortal. Sci Signal.

