# Supplementary figures and images for: A single-cell survey of cellular hierarchy in acute myeloid leukemia
Source: J Hematol Oncol. 2020 Sep 25;13:128. doi: 10.1186/s13045-020-00941-y (PMC7517826; doi:10.1186/s13045-020-00941-y)

**A**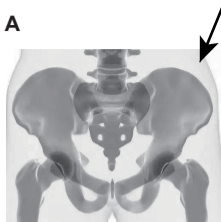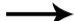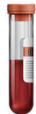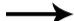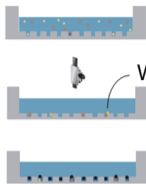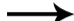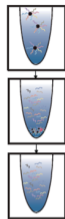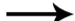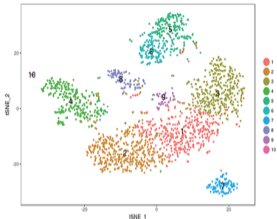**B**

N01

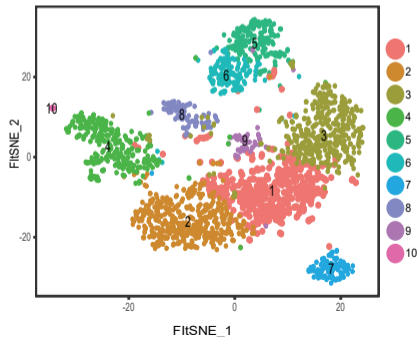

N02

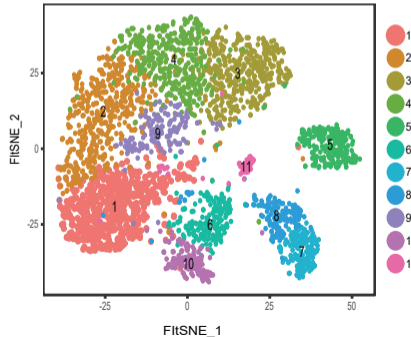

N03

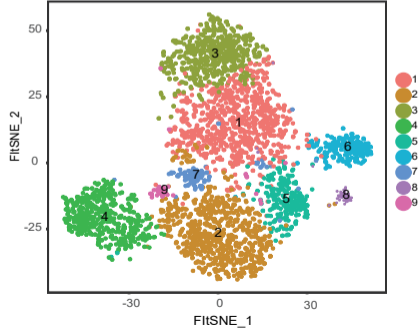

Supplement: Supplementary file 1 — Additional file 1: Fig. S1. Workflow and Individual t-SNE maps of normal donors. [file 13045_2020_941_MOESM1_ESM.pdf]

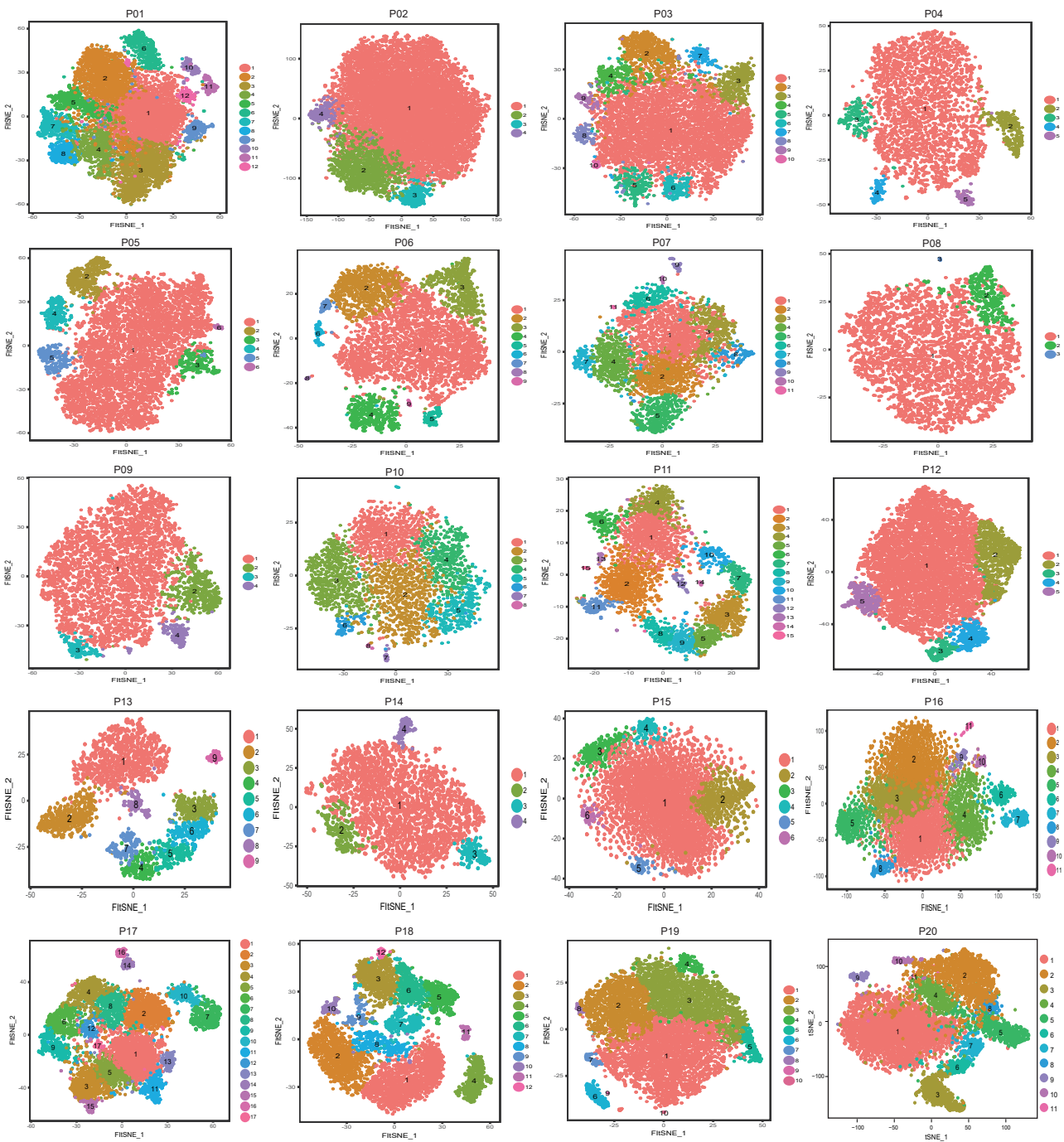

Supplement: Supplementary file 3 — Additional file 3: Fig. S3. Individual t-SNE maps of P01-P20. [file 13045_2020_941_MOESM3_ESM.pdf]

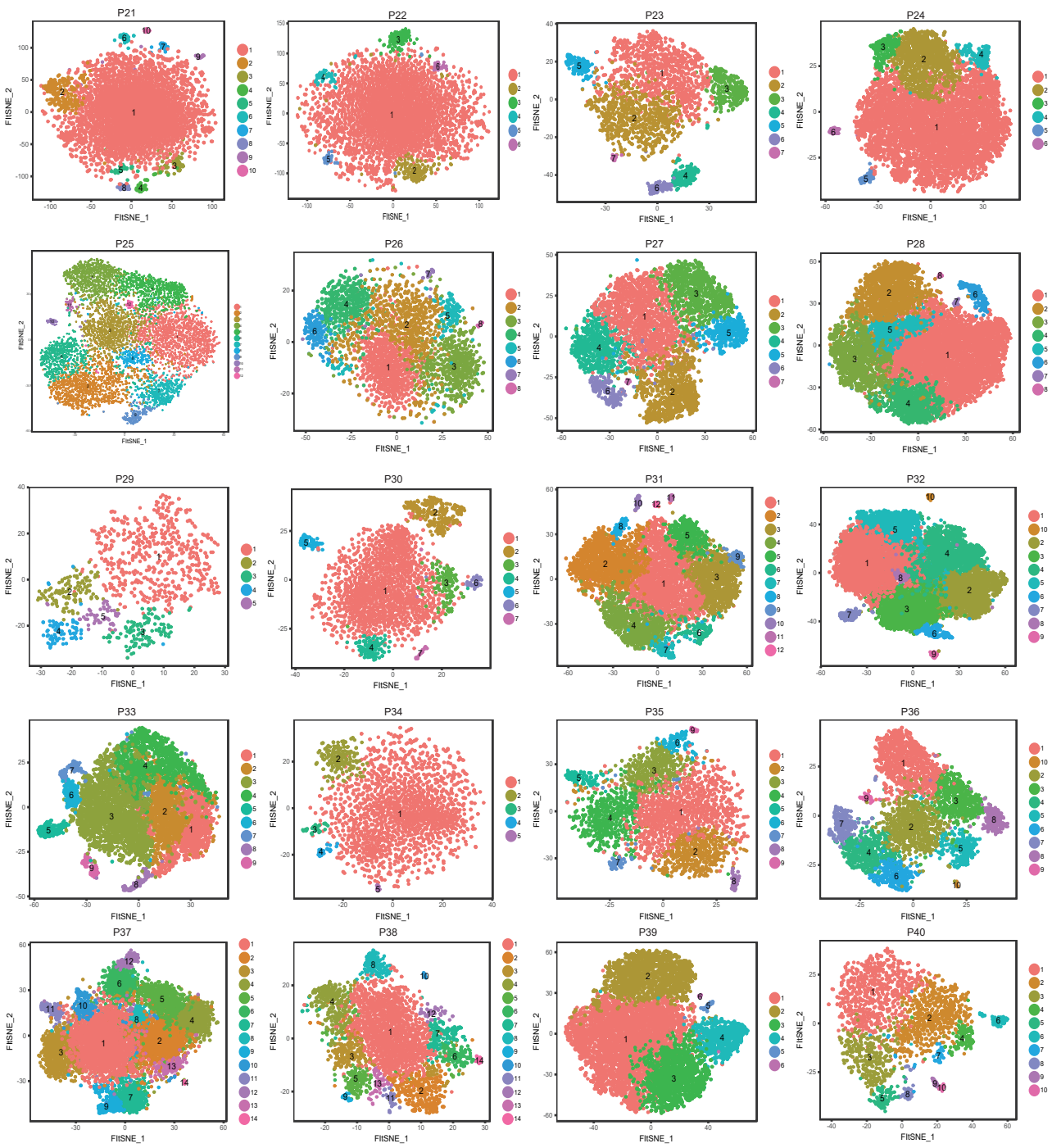

Supplement: Supplementary file 4 — Additional file 4: Fig. S4. Individual t-SNE maps of P21-P40. [file 13045_2020_941_MOESM4_ESM.pdf]

**A**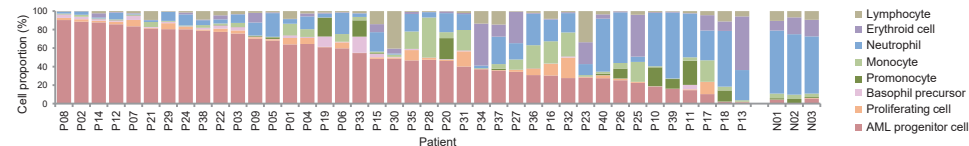**B**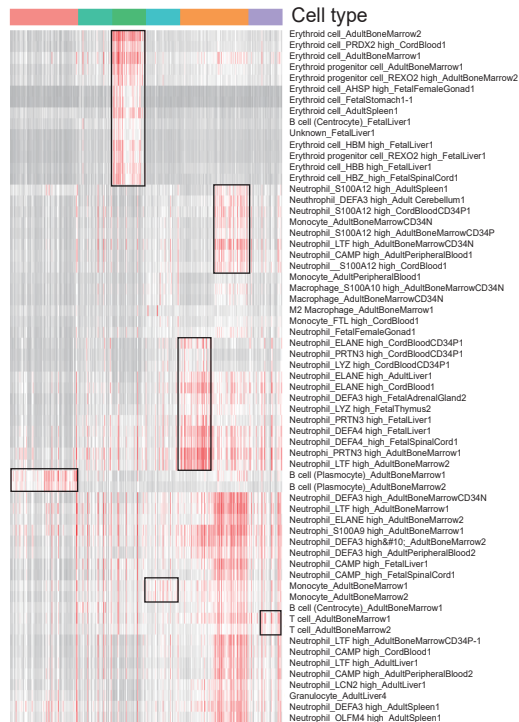**C**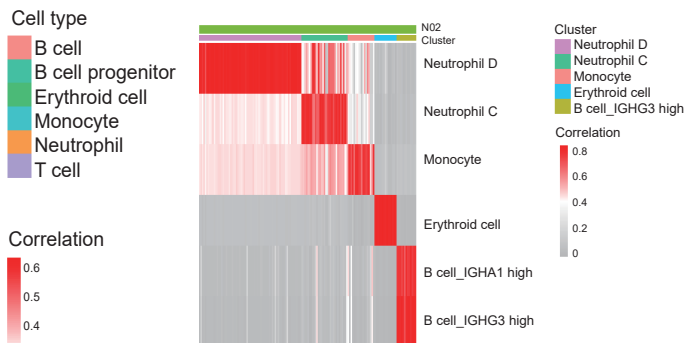**D**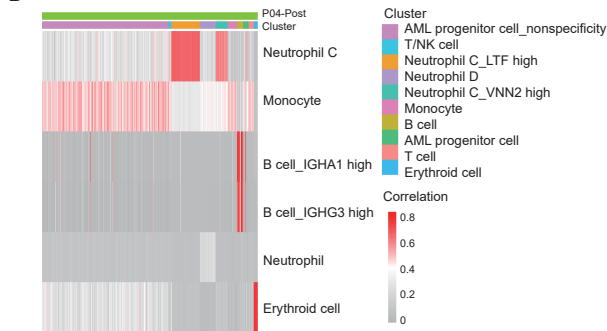

Supplement: Supplementary file 5 — Additional file 5: Fig. S5. Single-cell blast analysis. [file 13045_2020_941_MOESM5_ESM.pdf]

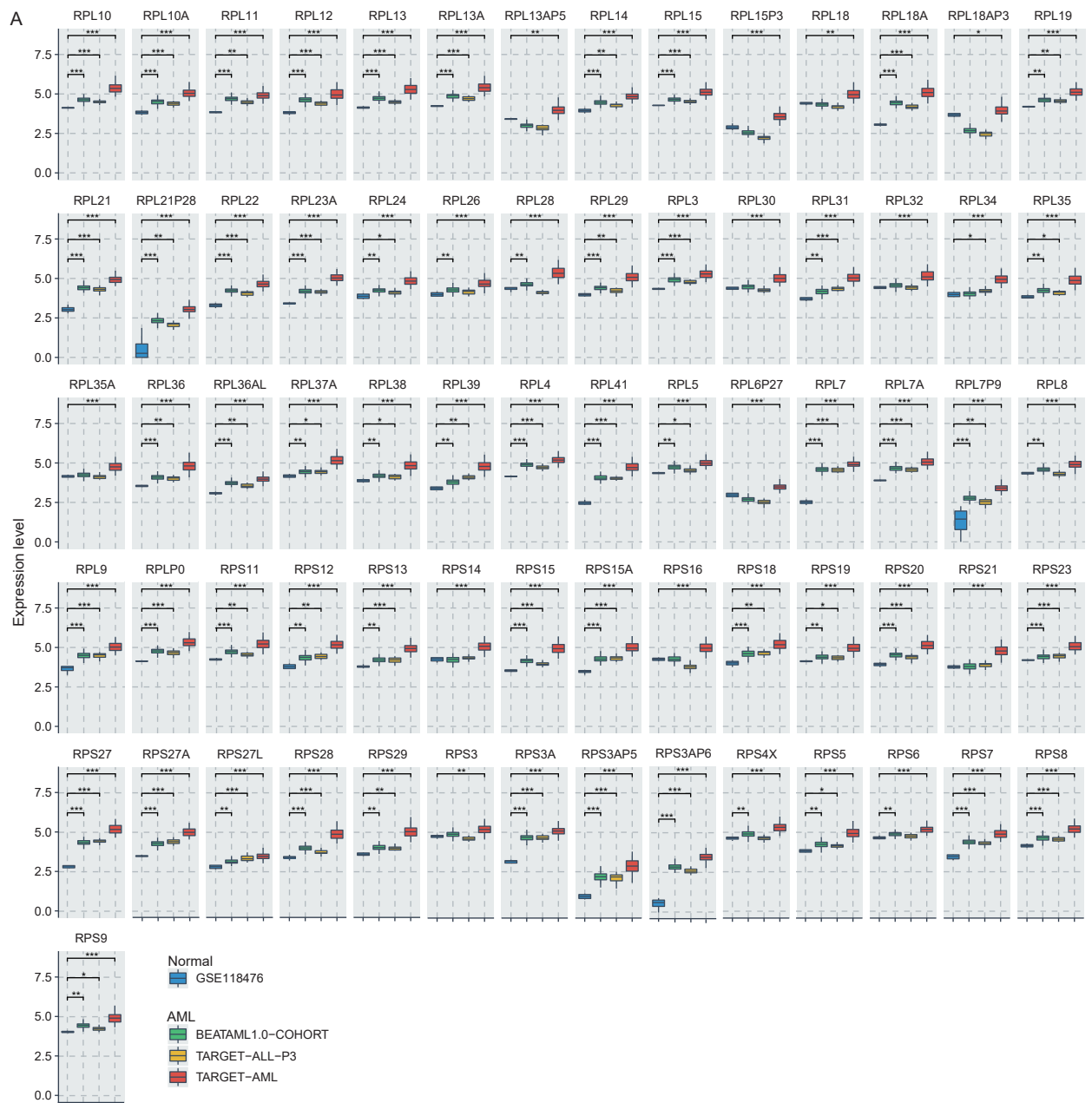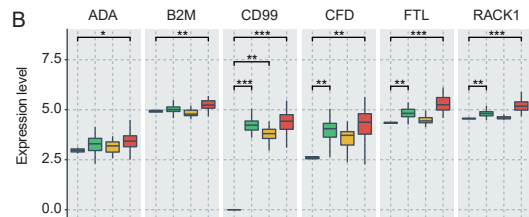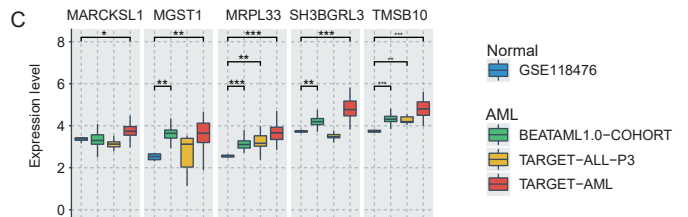

Supplement: Supplementary file 6 — Additional file 6: Fig. S6. Gene expression levels in TCGA. [file 13045_2020_941_MOESM6_ESM.pdf]

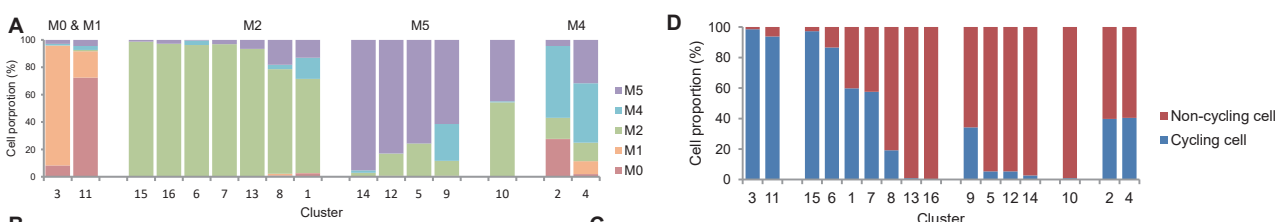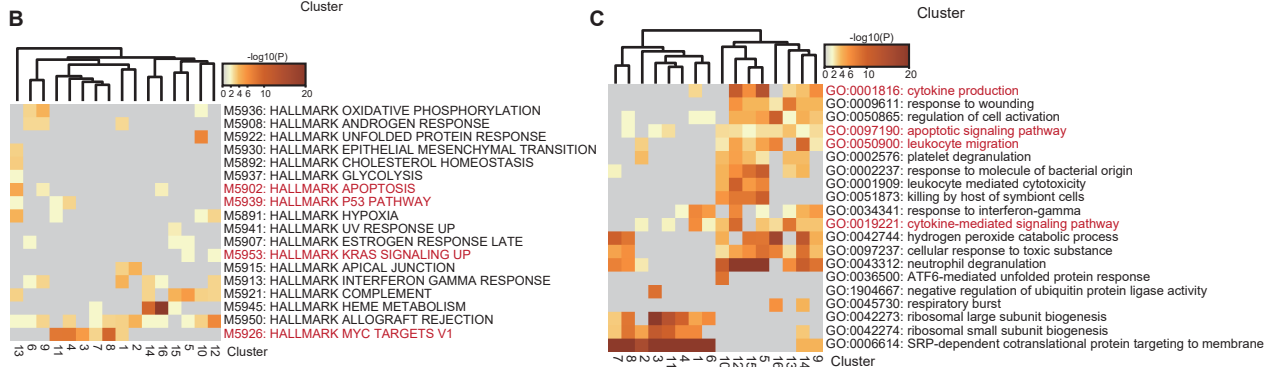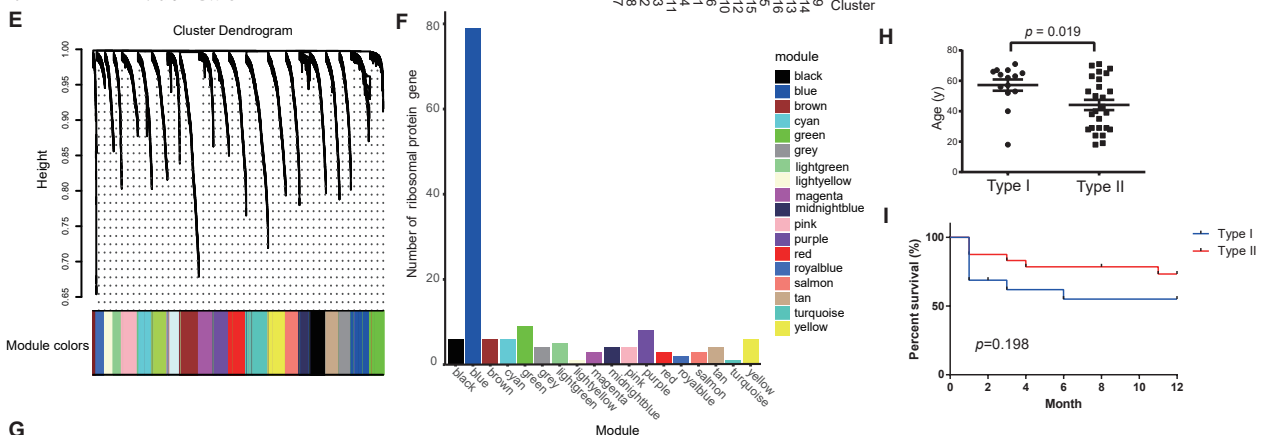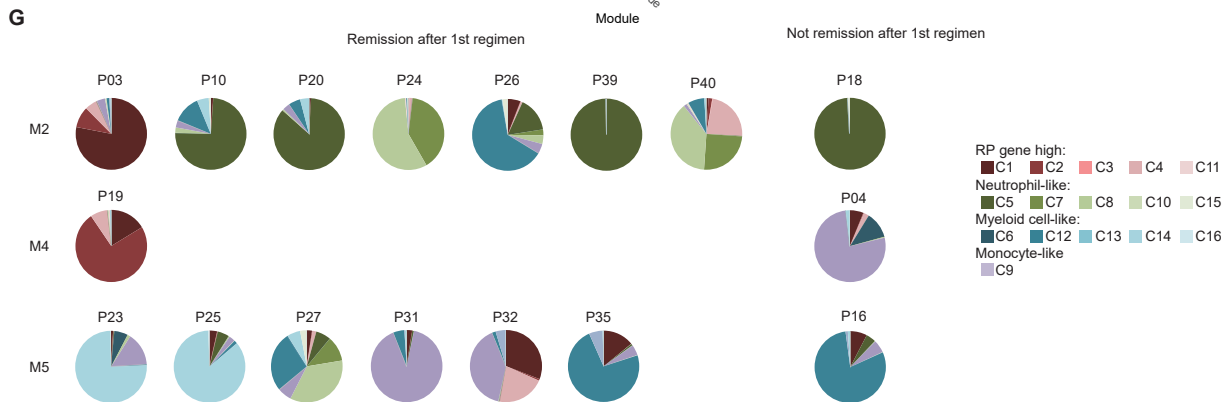

Supplement: Supplementary file 8 — Additional file 8: Fig. S8. Intratumoral heterogeneity in AML progenitor cells. [file 13045_2020_941_MOESM8_ESM.pdf]

P04-Post

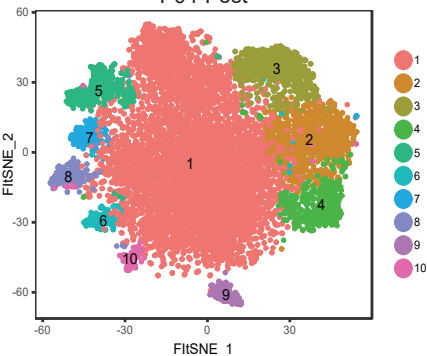

P20-Post

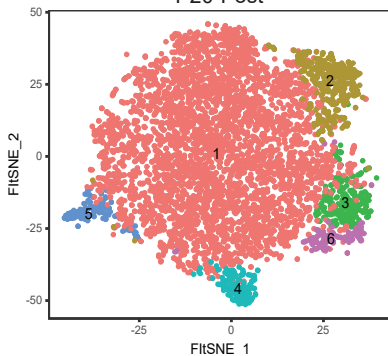

P-extra1-Pre

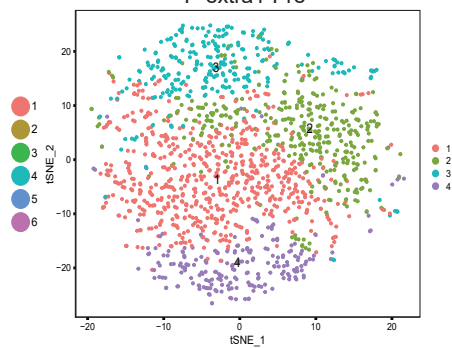

P-extra1-Post

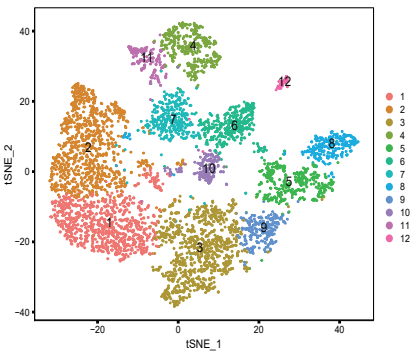

P-extra2-Pre

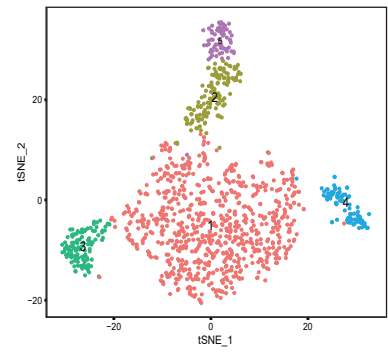

P-extra2-Post

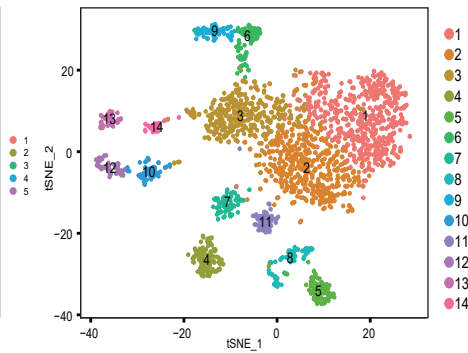

Supplement: Supplementary file 9 — Additional file 9: Fig. S9. Individual t-SNE maps of two extra patients and two patients post regimens. [file 13045_2020_941_MOESM9_ESM.pdf]

A

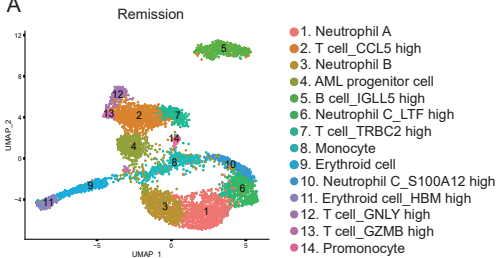

B

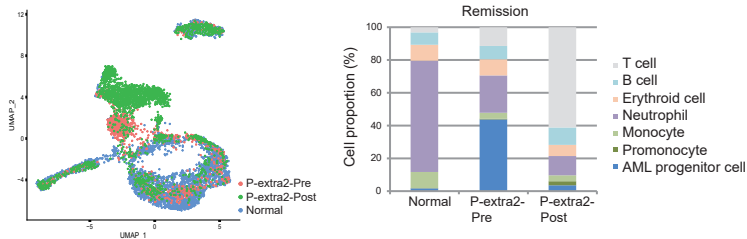

C

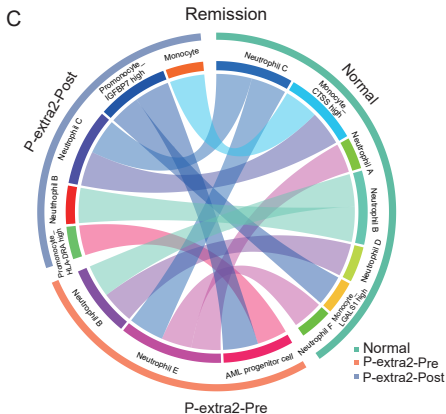

D

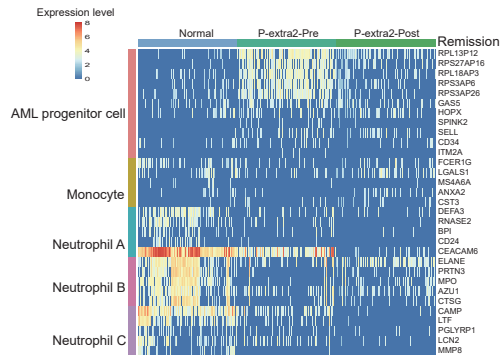

Supplement: Supplementary file 10 — Additional file 10: Fig. S10. Clinical implication of P-extra 2. [file 13045_2020_941_MOESM10_ESM.pdf]

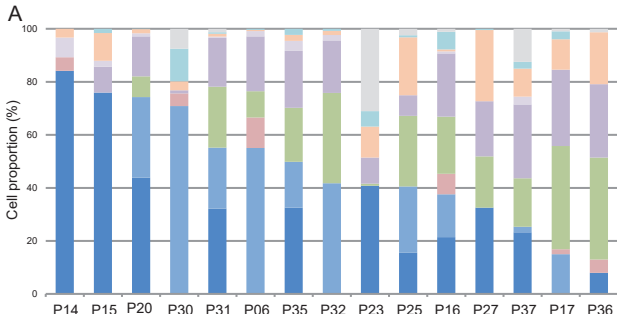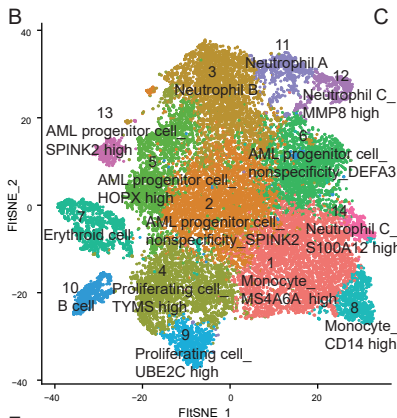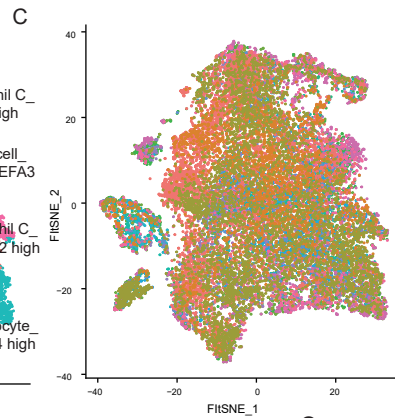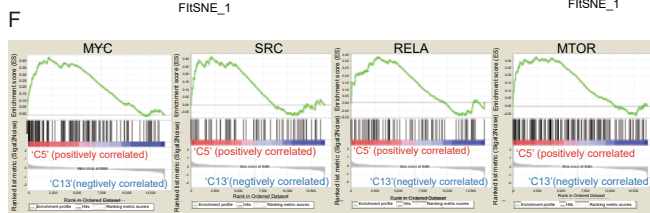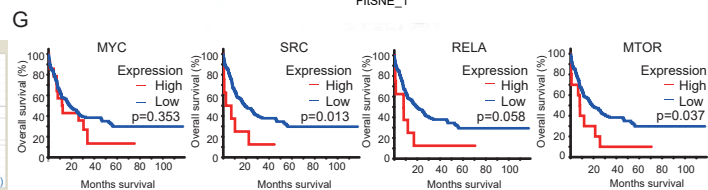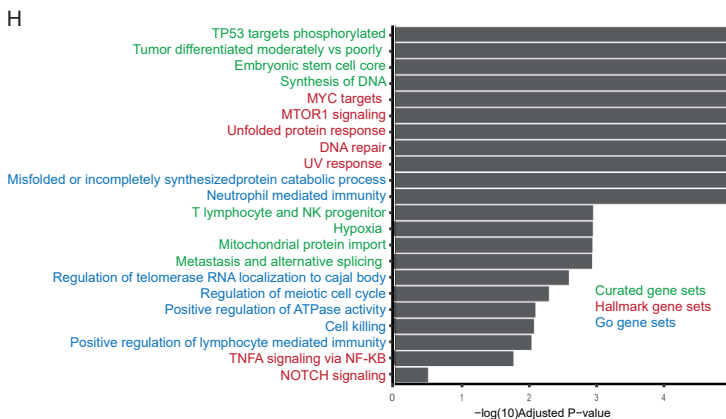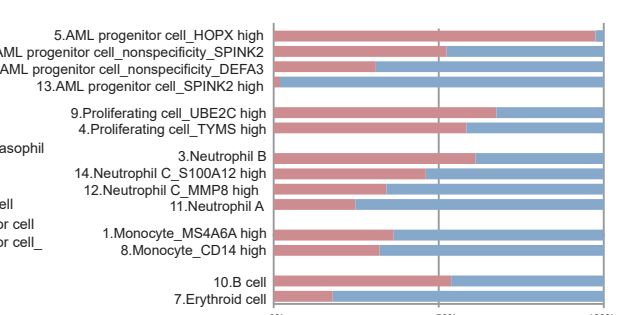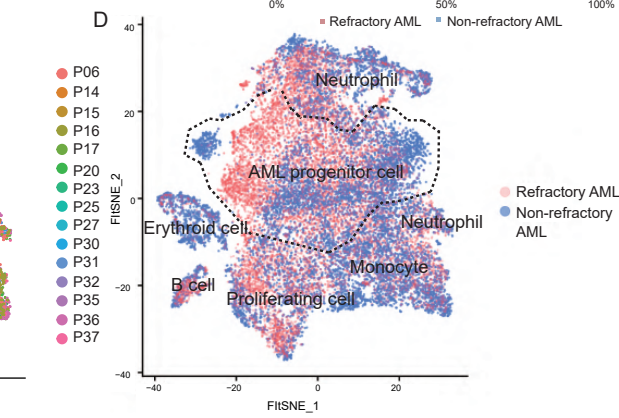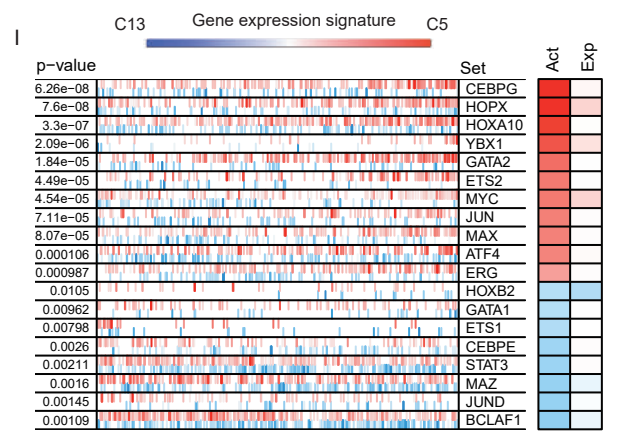

Supplement: Supplementary file 11 — Additional file 11: Fig. S11. Intratumoral heterogeneity in monocytic leukemia. [file 13045_2020_941_MOESM11_ESM.pdf]

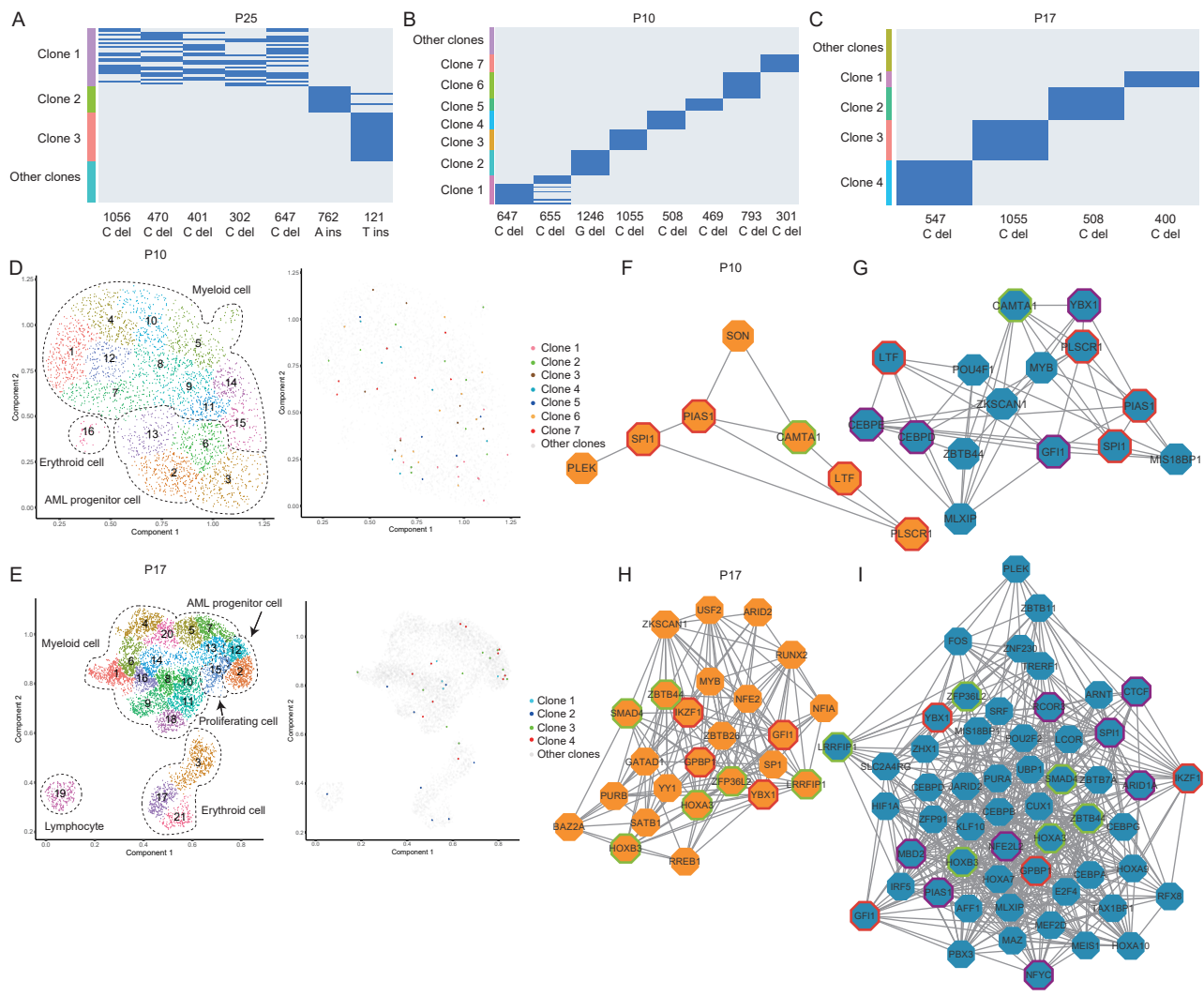

Supplement: Supplementary file 12 — Additional file 12: Fig. S12. Genetic mutation revealed by SMRT sequencing. [file 13045_2020_941_MOESM12_ESM.pdf]
