# Supplementary material for: A single-cell survey of cellular hierarchy in acute myeloid leukemia
Source: J Hematol Oncol. 2020 Sep 25;13:128. doi: 10.1186/s13045-020-00941-y (PMC7517826; doi:10.1186/s13045-020-00941-y)
Supplement: Supplementary file 2 — Additional file 2: Fig. S2. Neutrophil clusters and the marker genes. [file 13045_2020_941_MOESM2_ESM.pdf]

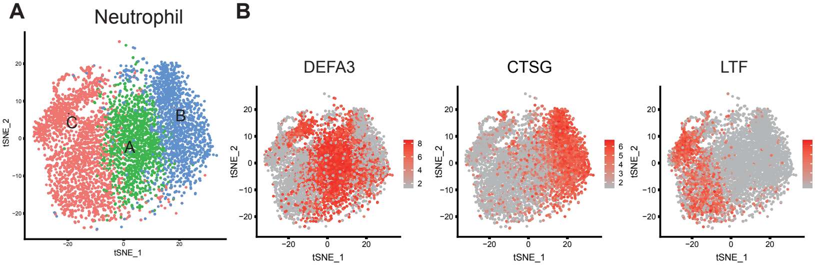

C

Cell type	Marker gene
Neutrophil A	DEFA3, BPI, CEACAM6, CD24, RNASE2
Neutrophil B	CTSG, PRTN3, MPO, ELANE, AZU1
Neutrophil C	MMP8, CAMP, LTF, PGLYRP1, LCN2
Neutrophil D	Combination of Neutrophil A & B
Neutrophil E	Combination of Neutrophil A & C
Neutrophil F	Combination of Neutrophil A, B & C
