# Supplementary material for: A single-cell survey of cellular hierarchy in acute myeloid leukemia
Source: J Hematol Oncol. 2020 Sep 25;13:128. doi: 10.1186/s13045-020-00941-y (PMC7517826; doi:10.1186/s13045-020-00941-y)
Supplement: Supplementary file 7 — Additional file 7: Fig. S7. Metascape enrichment network, VIPER and FLT3 analyses. [file 13045_2020_941_MOESM7_ESM.pdf]

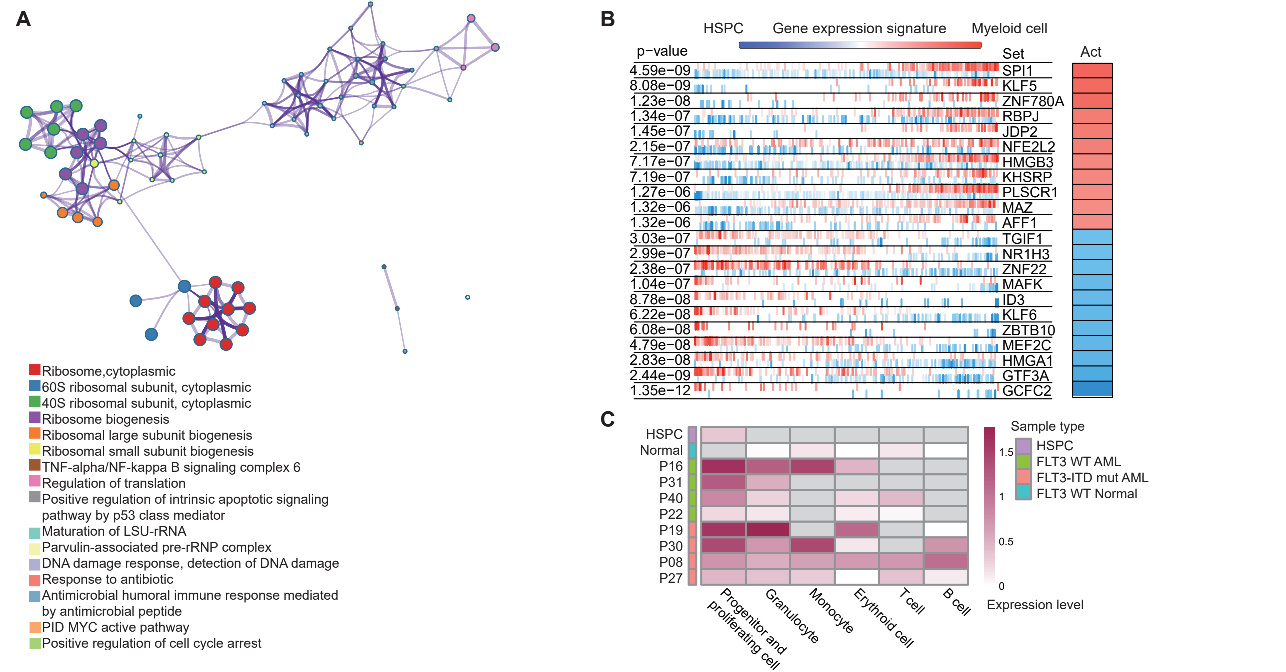
A
Ribosome,cytoplasmic
60S ribosomal subunit, cytoplasmic
40S ribosomal subunit, cytoplasmic
Ribosome biogenesis
Ribosomal large subunit biogenesis
Ribosomal small subunit biogenesis
TNF-alpha/NF-kappa B signaling complex 6
Regulation of translation
Positive regulation of intrinsic apoptotic signaling pathway by p53 class mediator
Maturation of LSU-rRNA
Parvulin-associated pre-rRNP complex
DNA damage response, detection of DNA damage
Response to antibiotic
Antimicrobial humoral immune response mediated by antimicrobial peptide
PID MYC active pathway
Positive regulation of cell cycle arrest
B
HSPC
Gene expression signature
Myeloid cell
p−value
Set
Act
4.59e−09 SPI1
8.08e−09 KLF5
1.23e−08 ZNF780A
1.34e−07 RBPJ
1.45e−07 JDP2
2.15e−07 NFE2L2
7.17e−07 HMGB3
7.19e−07 KHSRP
1.27e−06 PLSCR1
1.32e−06 MAZ
1.32e−06 AFF1
3.03e−07 TGIF1
2.99e−07 NR1H3
2.38e−07 ZNF22
1.04e−07 MAFK
8.78e−08 ID3
6.22e−08 KLF6
6.08e−08 ZBTB10
4.79e−08 MEF2C
2.83e−08 HMGA1
2.44e−09 GTF3A
1.35e−12 GCFC2
C
HSPC
Normal
P16
P31
P40
P22
P19
P30
P08
P27
Progenitor and proliferating cell
Granulocyte
Monocyte
Erythroid cell
T cell
B cell
1.5
1
0.5
0
Expression level
Sample type
HSPC
FLT3 WT AML
FLT3-ITD mut AML
FLT3 WT Normal
