# Supplementary material for: A single-cell survey of cellular hierarchy in acute myeloid leukemia
Source: J Hematol Oncol. 2020 Sep 25;13:128. doi: 10.1186/s13045-020-00941-y (PMC7517826; doi:10.1186/s13045-020-00941-y)
Supplement: Supplementary file 13 — Additional file 13: Fig. S13. Target searching based on the HCL. [file 13045_2020_941_MOESM13_ESM.pdf]

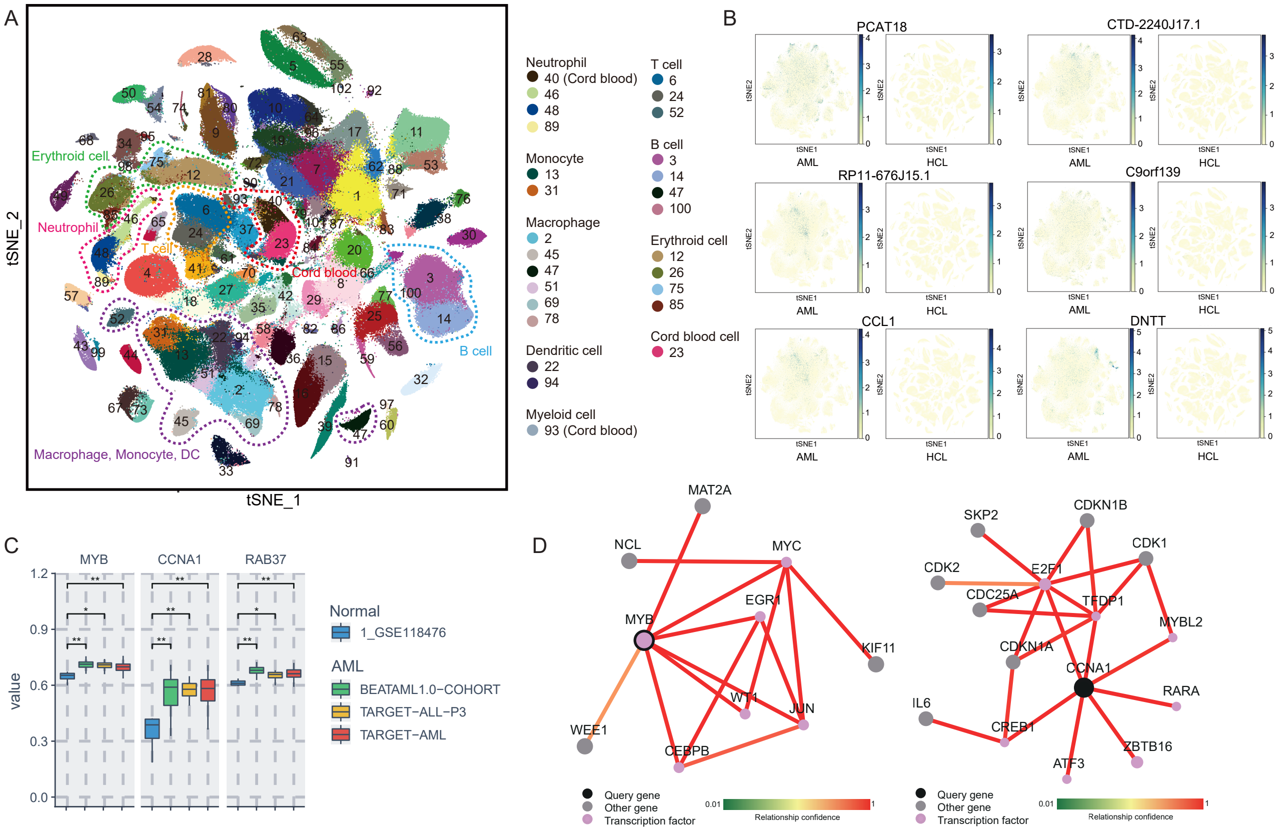

A
B
C
D
Erythroid cell
Neutrophil
T cell
Cord blood
B cell
Macrophage, Monocyte, DC
tSNE_1
tSNE_2
Neutrophil
40 (Cord blood)
46
48
89
Monocyte
13
31
Macrophage
2
45
47
51
69
78
Dendritic cell
22
94
Myeloid cell
93 (Cord blood)
T cell
6
24
52
B cell
3
14
47
100
Erythroid cell
12
26
75
85
Cord blood cell
23
PCAT18
CTD-2240J17.1
RP11-676J15.1
C9orf139
CCL1
DNTT
AML
HCL
MYB
CCNA1
RAB37
value
Normal
1_GSE118476
AML
BEATAML1.0−COHORT
TARGET−ALL−P3
TARGET−AML
MAT2A
NCL
MYC
EGR1
MYB
KIF11
WT1
JUN
WEE1
CEBPB
SKP2
CDKN1B
CDK1
CDK2
E2F1
CDC25A
TFDP1
MYBL2
CDKN1A
CCNA1
RARA
IL6
CREB1
ZBTB16
ATF3
Query gene
Other gene
Transcription factor
Relationship confidence
